# Supplementary material for: A phenome-wide association and Mendelian Randomisation study of polygenic risk for depression in UK Biobank
Source: Nat Commun. 2020 May 8;11:2301. doi: 10.1038/s41467-020-16022-0 (PMC7210889; doi:10.1038/s41467-020-16022-0)
Supplement: Supplementary file 16 — Reporting Summary [file 41467_2020_16022_MOESM16_ESM.pdf]

nature research

Corresponding author(s): Xueyi Shen, and Andrew M McIntosh

Last updated by author(s): Mar 19, 2020

Reporting Summary

Nature Research wishes to improve the reproducibility of the work that we publish. This form provides structure for consistency and transparency in reporting. For further information on Nature Research policies, see Authors & Referees and the Editorial Policy Checklist .

Statistics

For all statistical analyses, confirm that the following items are present in the figure legend, table legend, main text, or Methods section.

n/a	Confirmed	
☐	☒	The exact sample size (*n*) for each experimental group/condition, given as a discrete number and unit of measurement
☐	☒	A statement on whether measurements were taken from distinct samples or whether the same sample was measured repeatedly
☐	☒	The statistical test(s) used AND whether they are one- or two-sided<br>*Only common tests should be described solely by name; describe more complex techniques in the Methods section.*
☐	☒	A description of all covariates tested
☐	☒	A description of any assumptions or corrections, such as tests of normality and adjustment for multiple comparisons
☐	☒	A full description of the statistical parameters including central tendency (e.g. means) or other basic estimates (e.g. regression coefficient) AND variation (e.g. standard deviation) or associated estimates of uncertainty (e.g. confidence intervals)
☐	☒	For null hypothesis testing, the test statistic (e.g. *F*, *t*, *r*) with confidence intervals, effect sizes, degrees of freedom and *P* value noted<br>*Give P values as exact values whenever suitable.*
☒	☐	For Bayesian analysis, information on the choice of priors and Markov chain Monte Carlo settings
☒	☐	For hierarchical and complex designs, identification of the appropriate level for tests and full reporting of outcomes
☐	☒	Estimates of effect sizes (e.g. Cohen's *d*, Pearson's *r*), indicating how they were calculated

Our web collection on statistics for biologists contains articles on many of the points above.

Software and code

Policy information about availability of computer code

Data collection	No software used for data collection
Data analysis	R version 3.2.3 and version 3.3.2<br>RStudio version 0.98.1080<br>PRSice version 2.0<br>PLINK version 1.9<br>BGENIE version 1 (for depression GWAS used in Howard et al. analysis) and version 3 (for GWAS on neuroimaging variables)<br>METAL version 2011-03-25<br>Linkage Disequilibrium Score Regression v1.0.0<br>R package 'stats' version 3.2.3 (under R v3.2.3)<br>R package 'nlme' version 3.1.131 (under R v3.2.3)<br>R package 'TwoSampleMR' version 0.4.22 (under R v3.3.2)<br>R package 'MRPRESSO' version 1.0 (under R v3.3.2)<br>R package 'lavaan' version 0.5.23.1097 (under R v3.2.3)

For manuscripts utilizing custom algorithms or software that are central to the research but not yet described in published literature, software must be made available to editors/reviewers. We strongly encourage code deposition in a community repository (e.g. GitHub). See the Nature Research guidelines for submitting code & software for further information.

Data

Policy information about availability of data

All manuscripts must include a data availability statement. This statement should provide the following information, where applicable:

- Accession codes, unique identifiers, or web links for publicly available datasets
- A list of figures that have associated raw data
- A description of any restrictions on data availability

The data used in the present study is available from UK Biobank with restrictions applied. Data was used under license, and thus not publicly available. Access to the UK Biobank data can be requested through a standard protocol (https://www.ukbiobank.ac.uk/register-apply/). The summary statistics of PGC_139k can be accessed from https://doi.org/10.7488/ds/2458. A data transfer agreement is required for accessing the 23andMe_307k. summary statistics for the GWAS of depression (https://research.23andme.com/dataset-access/).

Field-specific reporting

Please select the one below that is the best fit for your research. If you are not sure, read the appropriate sections before making your selection.

☒ Life sciences ☐ Behavioural & social sciences ☐ Ecological, evolutionary & environmental sciences

For a reference copy of the document with all sections, see nature.com/documents/nr-reporting-summary-flat.pdf

Life sciences study design

All studies must disclose on these points even when the disclosure is negative.

Sample size	No statistical method was used to predetermine the sample sizes. The latest data releases from UK Biobank Imaging study were used in the current analyses (released in two waves in May and October 2018).
Data exclusions	We removed data from participants in the testing samples that are related within the testing samples, related with individuals in the training dataset, therefore excluding confounding effects introduced by family structures. Participants with an non-European ancestry were removed, in order to remain consistent with the training sample which was consist of Caucasian participants.<br>In total, 6,951 individuals were removed from current analyses (3,353 removed from the discovery sample and 3,598 from the replication sample). Related individuals were identified using the KING's criteria (http://people.virginia.edu/~wc9c/KING/manual.html). Non-European ancestry was identified based on k-means clustering on the genetic principal components. Individuals were further removed if they showed heterogeneous genotype frequencies, Hardy-Weinberg disequilibrium (p<10e-5), low minor allele frequency (<0.01), low imputation accuracy (<0.1), low call rate (<95%) and high missingness (>95%).<br>Behavioural phenotypes were excluded if N<2,000 in the discovery sample. This was to remove phenotypes that were likely to suffer from a lack of statistical power.<br>For neuroimaging phenotypes, individuals who have scores for the first unrotated principal component outside of +/-3 standard deviation from mean were excluded for analysis on diffusion tensor imaging data. This approach is to control the effect driven by outliers, which is consistent with previous work (doi.org/10.1016/j.bpsc.2018.07.006). After this step, diffusion tensor imaging data for fractional anisotropy from 110 individuals were excluded (49 and 61 from the discovery and replication samples, respectively), and for mean diffusivity, data from 142 individuals were excluded (73 and 69 from the discovery and replication samples, respectively).
Replication	Data from 21,888 individuals who participated in the UK Biobank imaging study were included in the current study (released in two waves, in May and October 2018). The discovery sample included participants mainly from the first data release, and the replication sample from the second release (details for the discovery and replication samples can be found in Supplementary Figure 16. Significant associations found in the discovery sample were carried to be re-tested on the replication sample. Details can be found in the Results section in the main text. Replication was conducted once on the replication sample.
Randomization	UK Biobank is a population-based cohort. No experimental grouping was conducted.<br>In order to further remove confounding effects introduced by demographic variability, age, age^2, sex and MRI site were included as covariates for all analyses. Head position in the scanner were controlled for in the analyses on structural data. Mean motion within the scanner was corrected in the model for resting-state data. Intracranial volume was included as a covariate for the analysis on subcortical volumes. Hemisphere was controlled for in the analyses of neuroimaging variables if there is any bilateral measure.
Blinding	No experimental grouping was conducted. The data used in the current analyses were collected under standard protocols by the UK Biobank team, independent of the analyses.

Reporting for specific materials, systems and methods

We require information from authors about some types of materials, experimental systems and methods used in many studies. Here, indicate whether each material, system or method listed is relevant to your study. If you are not sure if a list item applies to your research, read the appropriate section before selecting a response.

Materials & experimental systems

n/a	Involved in the study	
☒	☐	Antibodies
☒	☐	Eukaryotic cell lines
☒	☐	Palaeontology
☒	☐	Animals and other organisms
☐	☒	Human research participants
☒	☐	Clinical data

Methods

n/a	Involved in the study	
☒	☐	ChIP-seq
☒	☐	Flow cytometry
☒	☐	MRI-based neuroimaging

Human research participants

Policy information about studies involving human research participants

Population characteristics	We used the latest releases from the UK Biobank Imaging project. The discovery sample included 10,674 individuals (age 45.9 - 80.3 years, mean=62.8, SD=7.4, 48.4% were men). The replication sample consisted of 11,214 individuals in total (age 46.5 - 80.8 years, mean age=64.4, SD=7.4, and 49.4% were men).
Recruitment	Participants were recruited from the overall UK Biobank population-based sample.
Ethics oversight	Data acquisition and analyses in the present study were conducted under UK Biobank Application #4844. Ethical approval was accepted by the National Health Service (NHS) Research Ethics Service (11/NW/0382).

Note that full information on the approval of the study protocol must also be provided in the manuscript.
