# Supplementary material for: A phenome-wide association and Mendelian Randomisation study of polygenic risk for depression in UK Biobank
Source: Nat Commun. 2020 May 8;11:2301. doi: 10.1038/s41467-020-16022-0 (PMC7210889; doi:10.1038/s41467-020-16022-0)
Supplement: Supplementary file 17 — Supplementary Information [file 41467_2020_16022_MOESM17_ESM.pdf]

Supplementary Information

A phenome-wide association study of polygenic risk scores for depression using behavioural and neuroimaging phenotypes from UK Biobank

Shen et al.
Division of Psychiatry, University of Edinburgh, Edinburgh, United Kingdom

Supplementary methods

The testing samples – the discovery and replication sample

The discovery sample included participants from the first data release (97% assessed at Cheadle, 3% at Newcastle) who were assessed in Cheadle, and included 10,674 individuals (age 45.9 - 80.3 years, mean=62.8, SD=7.4, 48.4% were men). The second release contained a higher proportion of individuals assessed in Newcastle (63% at Cheadle, 37% at Newcastle). The replication sample therefore included all participants included in the second release plus the smaller proportion (3%, N = 343) assessed at Newcastle in the first release. The replication sample consisted of 11,214 individuals in total (age 46.5 - 80.8 years, mean age=64.4, SD=7.4, and 49.4% were men, see Supplementary Figure 16).

Meta-analysis of GWAS on depression and the testing samples

Methods for the meta-analysis has been described in another paper by Howard et al. (2018) (see the main text). The only difference between the present paper and the paper cited above is the sample from UK Biobank. In the original meta-analysis paper, 371,435 unrelated, European-ancestry participants who have not participated in the PGC and 23andMe GWAS were included after genotyping quality check. The present paper further removed people who are related, have non-British ancestry, and those who attended imaging assessment, if there is a non-empty entry of 'date of attending assessment centre' in field 53.2.0 (http://biobank.ctsu.ox.ac.uk/crystal/field.cgi?id=53). A total of 22,404 people were left after this step. For the testing sample, which includes the discovery and replication samples, 516 participants were further removed for either having NA value reported in the MRI site field (f.54.2.0, http://biobank.ctsu.ox.ac.uk/crystal/field.cgi?id=54) or was recruited in Reading (code: 11026, http://biobank.ctsu.ox.ac.uk/crystal/coding.cgi?id=10), from which imaging-derived phenotypes were not yet available. This eventually leaves 21,888 people for the analyses conducted in the present study. GWAS was conducted on the subset of UK Biobank sample where people that have attended imaging assessment were excluded, using the BGENIE pipeline version 1[1], to keep consistent with the original GWAS conducted by Howard et al. (2018). Statistic models

and genotyping quality control remained the same as a depression GWAS conducted on UK Biobank. Meta-analysis was conducted using 'Metal'(version 2011-03-25)[2].

Generating depression-PGRS for the testing samples

DNA extraction and genotyping were described in the UK Biobank protocol documentation (http://www.ukbiobank.ac.uk/wp-content/uploads/2014/04/UKBiobank_genotyping_QC_documentation-web.pdf). In brief, sample collection was conducted by the UK Biobank team, and genotyping was conducted at the Affymetrix Research Services. SNP quality-control was conducted by the UK Biobank team based on the criterion of heterogeneous genotype frequencies in the same samples and across genotyping arrays, Hardy-Weinberg disequilibrium ($p<10^{-5}$), low minor allele frequency (<0.005), low imputation accuracy (<0.1) and low call rate (<95%). In addition to these steps, we further conducted sample control, removing participants who have missingness of >95%, have gender-mismatched genetic data, have non-European-ancestry and those who are related. Non-European ancestry was identified based on k-means clustering on the genetic principal components to identify white British ancestry[3] and those who declared non-white British ancestry were further removed. Relatedness was quantified by the kinship coefficient using the King's criteria[4]. First-degree relatives were identified as one family, and one of the participants in each family was randomly selected to add back into the sample to maximise sample size while ensuring relatedness removed.

Genotype data after quality check was then fed into PRSice 2.0 program. The clumping threshold was set as p=1, LD score r^2=0.25, distance threshold=250 Kb. P-thresholds for scoring were set at p<0.0005, p<0.001, p<0.005, p<0.01, p<0.05, p<0.1, p<0.5 and p<1. No phenotype data was fed into the program except for a dummy phenotype file that covers all the participants for the testing sample.

Supplementary information for behavioural phenotypes

Principal component analysis (PCA) was conducted on cognitive variables (Table 1 and Supplementary Table 18) to extract a measure of g of cognitive abilities. Tasks conducted at the

imaging assessment centres were used for this step because these measures cover much more people than online questionnaires (N~=20,000 for the former, and N~=11,000 for the latter). Variance explained by the first unrotated principal component was 30.2%.

Neuroimaging phenotypes in UK Biobank

T1 data was processed to estimate intracranial and subcortical volumes. First, total volumes for white matter, grey matter and peripheral cerebrospinal fluid were calculated, and the sum of the three was the derived intracranial volume. This derived intracranial volume is highly correlated with the field "Volumetric scaling from T1 head image to standard space" (f.25000.2.0) with a correlation coefficient of -0.898. Then volumes for thalamus, caudate, putamen, pallidum, hippocampus, amygdala, accumbens and brain stem (with 4th ventricle) were estimated. Participants with and estimated intracranial volume outside of +/-3 standard deviation from mean were excluded.

Diffusion tensor imaging (DTI) data pre-processing included correction for eddy currents and head motion, outlier-slices correction and grand distortion correction. Maps for fractional anisotropy (FA) and mean diffusivity (MD) were generated and FA maps were used to generate tract masks, using probabilistic tractography analysis by AutoPtx package from FSL[5]. Twenty-seven tracts were generated (12 bilateral and 3 unilateral tracts, see Supplementary Data 1)[6]. Weighted mean FA and MD were then calculated for each tract. Neurite Orientation Dispersion and Density Imaging (NODDI) measures were also generated as supplementary measures, which include neurite density (ICVF), volume fraction of isotropic diffusion (ISOVF) and orientation dispersion index (OD). To determine general variances in DTI measures and main subsets, as have validated in previous papers that weighted-mean DTI measures for major white matter tracts are highly correlated, which makes generating general variances possible, we performed PCA on (1) FA/MD/ICVF/ISOVF/OD of all 27 tracts (gTotal), (2) on association/commissural fibres (gAF), which connect the prefrontal cortex to other cortexes, (3) on thalamic radiations (gTR), consisted of tracts that link the thalamus to the cortex, and (4) on projection fibres (gPF), locating within brain stem or spinal cord or link them to the. Variance explained by the first latent component can be found in Supplementary Figure 20, and reports of correlation loadings of each variable on the latent

factors are in Supplementary Table 6. The above PCA was conducted on the total sample of both releases to achieve more accurate estimations. The scores for the first unrotated principal component were used as the indices for general variants of total variance and variances in three major subsets. In order to control for the effects driven by outliers, subjects with a gTotal for FA/MD/ICVF/ISOVF/OD outside of +/-3 standard deviation from mean were excluded (see Reference 26 in the main text).

Susceptibility weighted brain images were used to derive T2* measures[7,8]. White matter hyperintensity in the whole brain and in seven subcortical regions (accumbens, amygdala, caudate, hippocampus, pallidum, putamen and thalamus) were derived. Participants with whole-brain white matter hyperintensity or mean hyperintensity of subcortical regions outside of +/-3 standard deviation from mean were excluded.

Resting-state data was pre-processed through FSL-style motion correction, grand-mean intensity normalisation, high-pass temporal filtering, EPI unwarping and grand-distortion-correction unwarping. A group-level independent component analysis was conducted on the first 4,100 people to reduce data dimension[8]. The brain was therefore parcellated into 25 independent components, and 21 of them were left for further analyses after 4 discarded as being identified manually as noise components. The time-series data for nodes was then used to calculate functional connectivity between node pairs. It was achieved by estimating partial Pearson correlation with an L2 regularisation applied (rho set as 0.5 in FSLNets). All r-scores were then Fisher-transformed into z-scores. This resulted in a 21*21 correlation matrix of functional connectivity for each participant. In order to aid comprehension, all connectivity values were transformed into absolute strength by multiplying the sign of group-mean value for each of the connection. For amplitude of signal fluctuation, as the data has had high-pass temporal filtering, therefore the measure mainly represent temporal fluctuations of blood oxygen-level dependent signal[9]. An interactive website displaying group-mean maps for each node can be found in a URL: http://www.fmrib.ox.ac.uk/datasets/ukbiobank/group_means/rfMRI_ICA_d25_good_nodes.html. A connectome map of the nodes can be found in:
http://www.fmrib.ox.ac.uk/datasets/ukbiobank/netjs_d25/.

Sensitivity analysis of Mendelian Randomisation (MR) results

Standard visual inspection for MR results was conducted based on leave-one-out analysis (Supplementary Figures 7-11, produced using 'twosampleMR' R package version 0.4.22 under R 3.3.2). For the most robust results shown in Figure 5, all leave-one-out tests remained significant, indicating that the results were not driven by a single genetic variant, but by a consistent pattern among all variants.

Other than this, we have also conducted another sensitivity analysis, removing possible pleiotropic variants. We considered neuroticism as a major possible pleiotropic variable as it showed the strongest genetic correlation with depression in Howard et al.'s (2019) GWAS paper (see Supplementary Table 3 in the reference paper).

GWAS was conducted on the non-imaging sample in UK Biobank, using the neuroticism score created with the method identical to the study by Luciano et al[10]. Software, computational environment and quality check criteria were consistent with the GWAS for neuroimaging variables and the depression GWAS on UK Biobank (see main text). We also validated the summary statistics by calculating genetic correlation between this summary statistic and two other neuroticism GWAS[11,12] using LDhub[13]. The results are confirming (rg = 0.955 and 1.028, p for genetic correlation $< 6.07 \times 10^{-22}$). Genome-wide significant SNPs were clumped using the same clumping method for selecting genetic instruments for depression GWAS.

We then removed the genome-wide significant SNPs in depression GWAS in medium to high linkage disequilibrium (LD) with neuroticism (r>0.1). LD was extracted using R package LDlinkR (https://ldlink.nci.nih.gov/) for those SNPs on the same chromosome. European-ancestry reference panel from the 1000 Genomes Project was used (population code: CEU). Four genetic variants were thus removed (rs169235, rs30266, rs4969391 and rs7837935). Another MR was conducted on the remaining genetic variants to test the causal relationship of depression -> brain, the results remained consistent with the initial findings. For the most robust findings reported in Figure 5, all remained significant in at least two MR methods (see Supplementary Table 7).

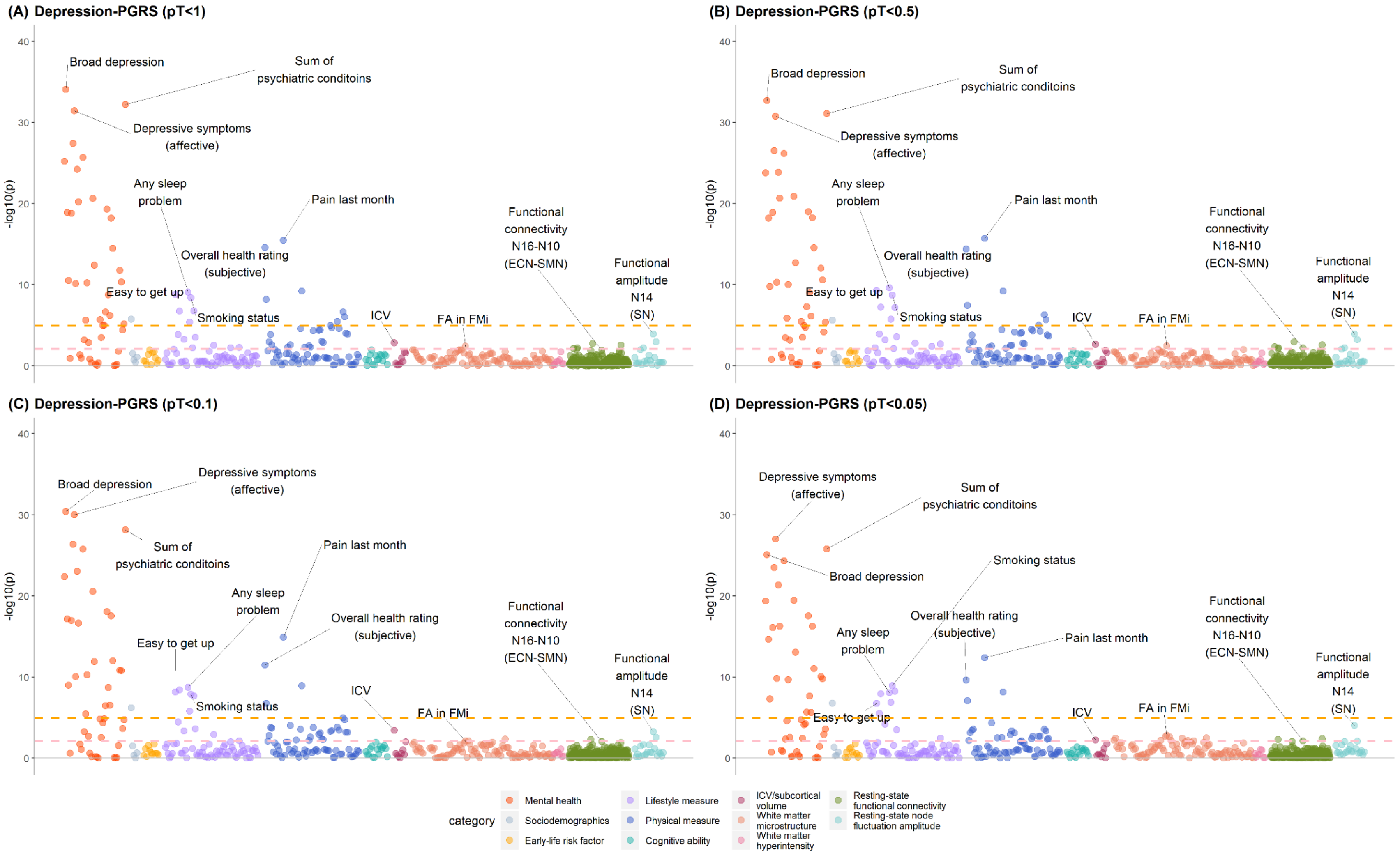
(A) Depression-PGRS (pT<1)
(B) Depression-PGRS (pT<0.5)
(C) Depression-PGRS (pT<0.1)
(D) Depression-PGRS (pT<0.05)
-log10(p)
0
10
20
30
40
Broad depression
Sum of psychiatric conditoins
Depressive symptoms (affective)
Any sleep problem
Pain last month
Overall health rating (subjective)
Easy to get up
Smoking status
ICV
FA in FMi
Functional connectivity N16-N10 (ECN-SMN)
Functional amplitude N14 (SN)
category
Mental health
Sociodemographics
Early-life risk factor
Lifestyle measure
Physical measure
Cognitive ability
ICV/subcortical volume
White matter microstructure
White matter hyperintensity
Resting-state functional connectivity
Resting-state node fluctuation amplitude

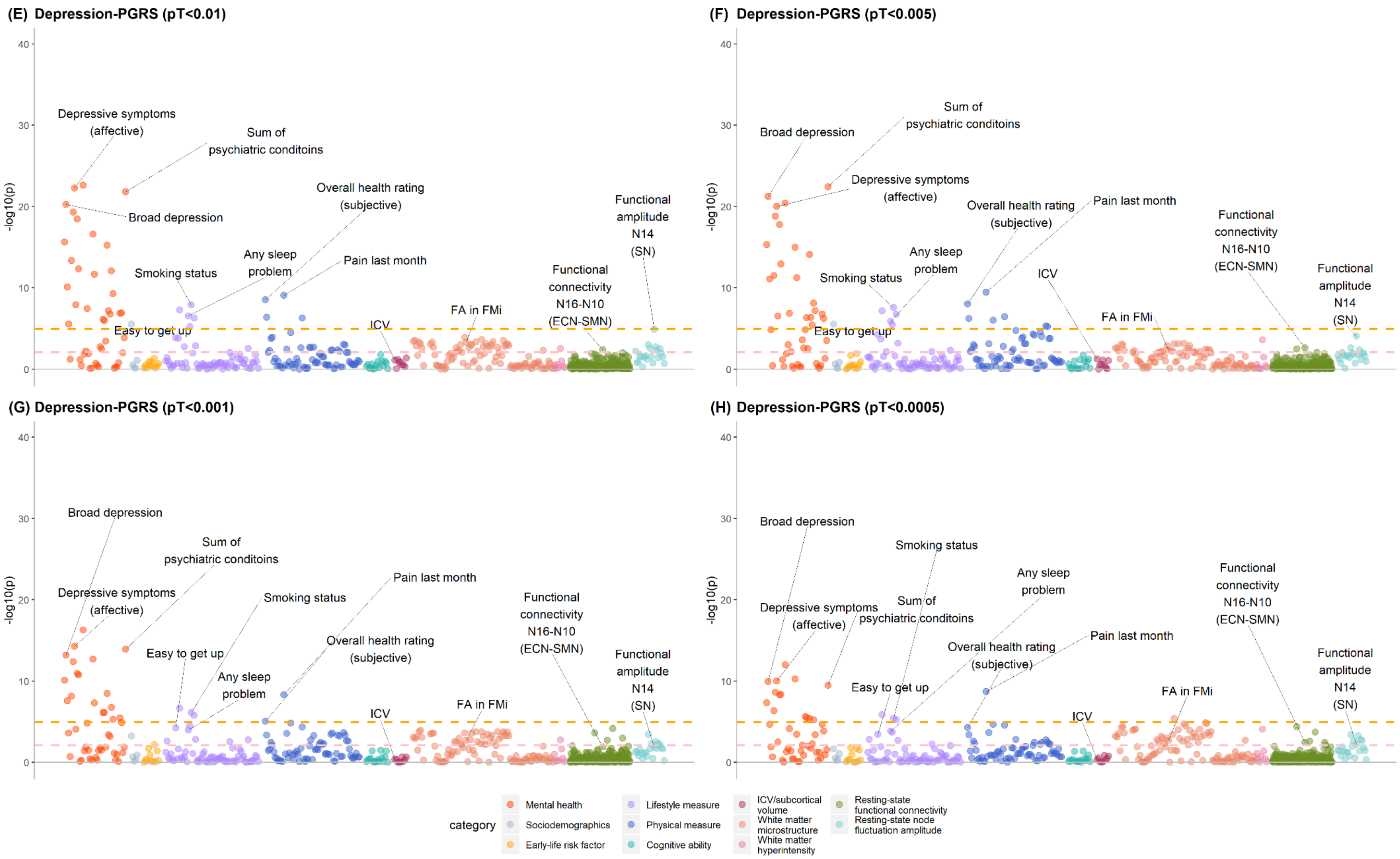

Supplementary Figure 1. Significance plots for PheWAS results on all depression-PRS p-thresholds for generating the scores (pT). Panels (A-D) are shown on this page and the other panels (E-H) are shown on the next page. The x axes represent phenotypes, and the y axes represent the -$\log_{10}$ of uncorrected p values of two-sided test for linear regression between depression-PRS and each of the phenotype. Each dot represents one phenotype, and the colours indicate their according categories. Top associations found in depression-PGRS at pT<1 and pT<0.01 (see the main text) are annotated in each panel. The pink dashed line indicates the threshold for FDR-corrected significance. The orange dashed line indicates the threshold for Bonferroni-corrected significance.

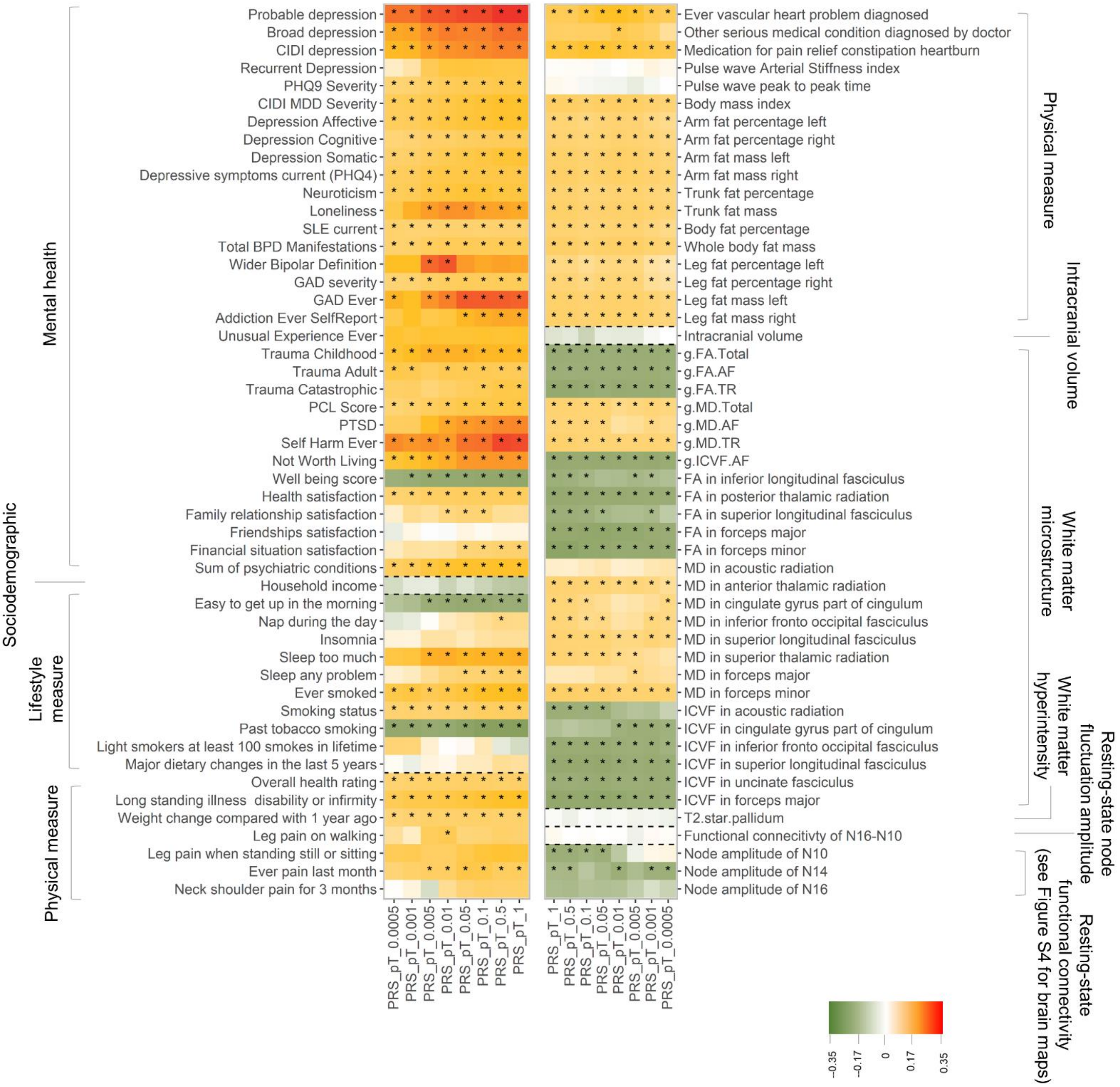

Supplementary Figure 2. Heatmap for results from the replication sample. Traits were selected based on whether they were associated with depression-PGRS at four p-thresholds minimal in the discovery sample. Shades of cells indicate the standardised effect sizes (β) for the linear regression between depression-PRS and each phenotype. A larger effect size was shown by a darker colour. Cells with an asterisk were significant after FDR-correction.

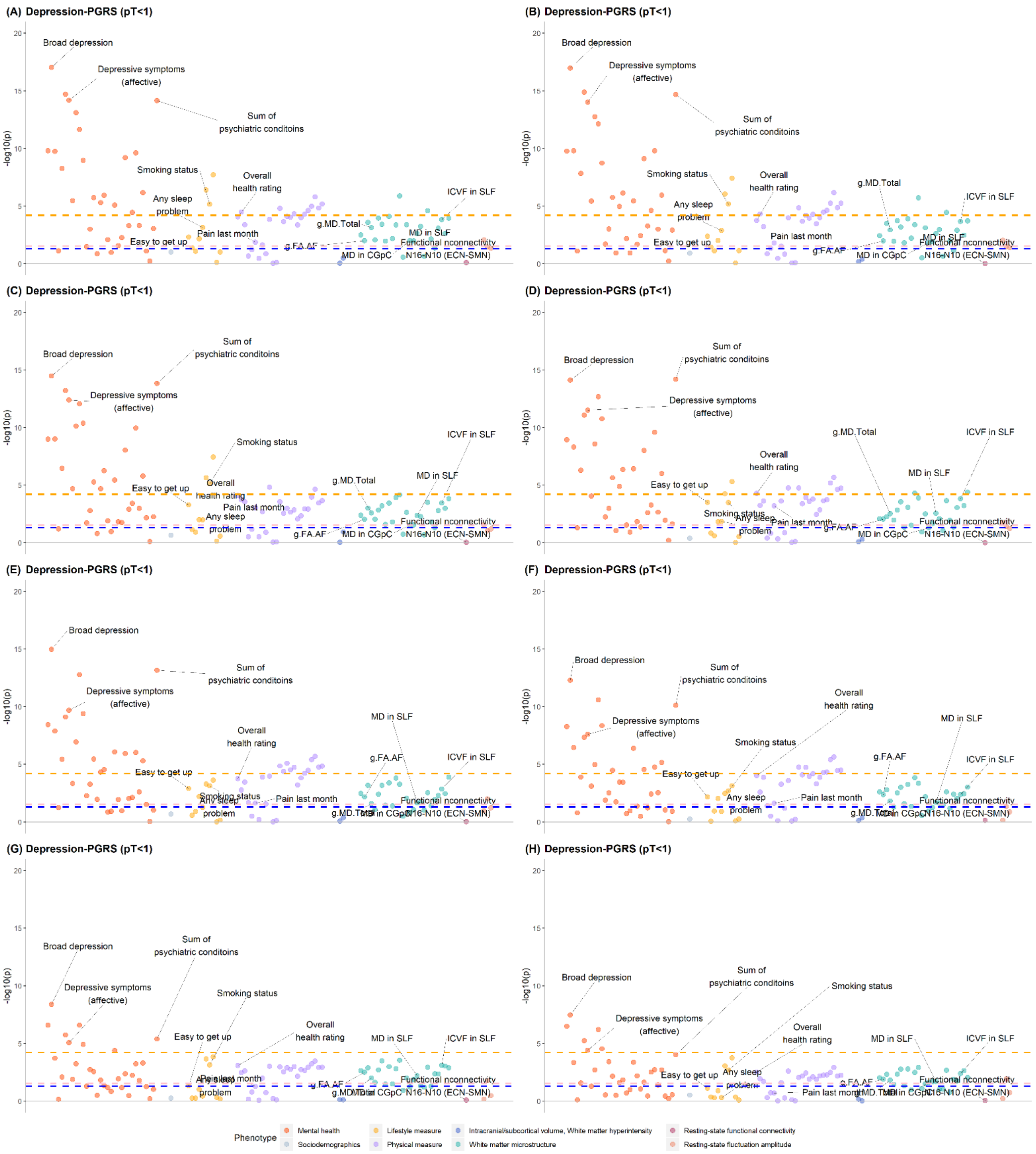

Supplementary Figure 3. Significance maps for replication analysis on all depression-PGRS on eight p-thresholds (pT) for generating the scores. In the replication analysis, traits that were significantly associated with depression-PGRS at minimal two pT were selected. The selected traits were tested in the replication sample at all eight pT. These tests were then FDR-corrected altogether (see methods). Top associations found in the discovery sample were annotated in each panel. Although the p-value maps for depression-PGRS pT<1 and pT<0.01 are presented in Figure 3, here for the purpose of completeness, figures for these two pT are presented again below. The x axes represent phenotypes, and the y axes represent the -$\log_{10}$ of uncorrected p values of two-sided test for linear regression between depression-PRS and each of the phenotype. Each dot represents one phenotype, and the colours indicate their according categories. As it is replication test, we added an extra dashed line in the figures to indicate nominally significance. The blue dashed lines in the panels represent the nominally significance threshold (p=0.05), the pink dashed lines represent the FDR-corrected significance threshold (q_{FDR}=0.05), and the orange dashed lines represent the Bonferroni-corrected significance threshold.

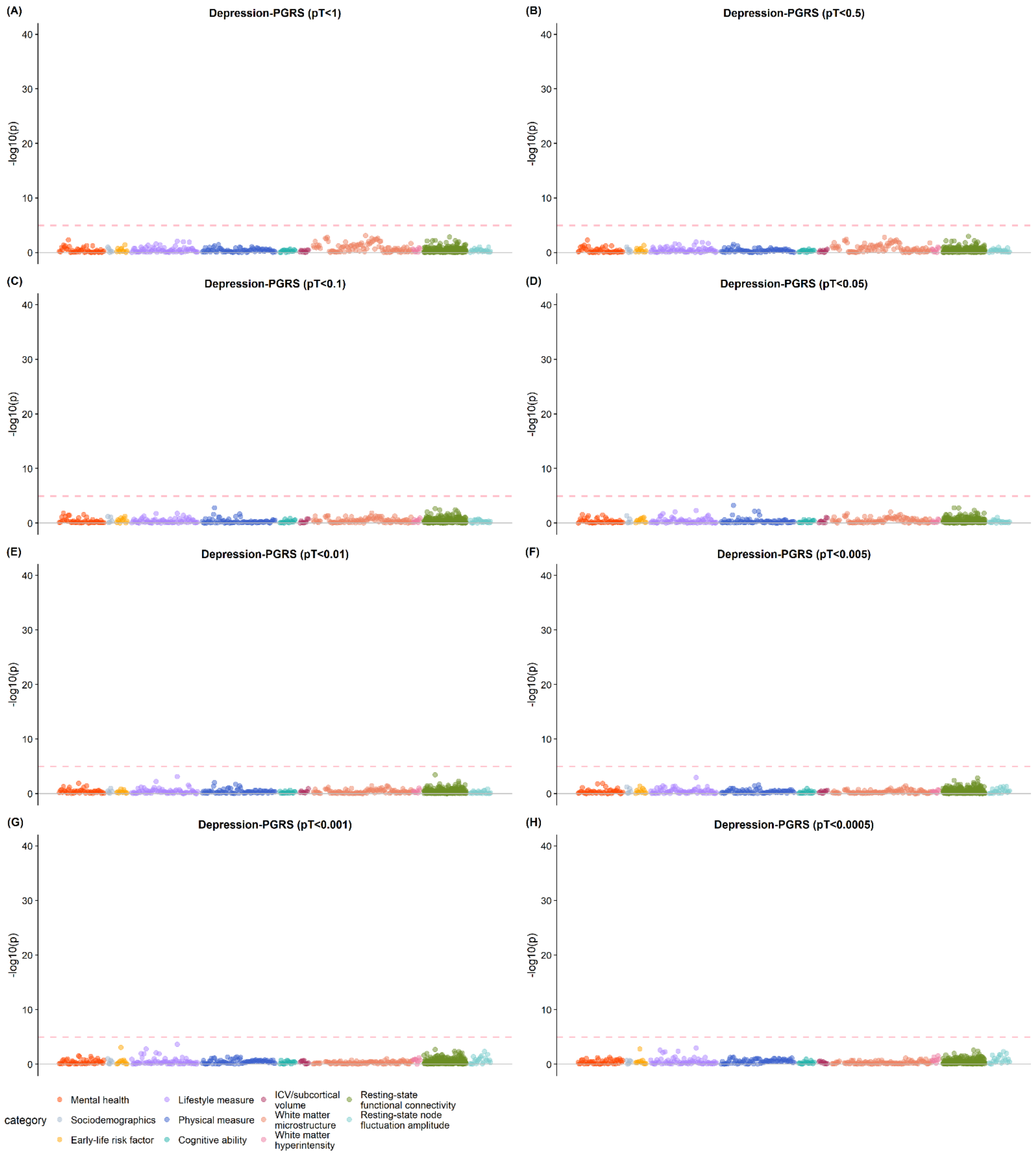

Supplementary Figure 4. Significance maps for the interaction term of MRI site × depression-PGRS for the traits that were found associated with depression-PGRS at minimal four p-thresholds for generating the scores. The x axes represent phenotypes, and the y axes represent the -$\log_{10}$ of uncorrected p values of two-sided test for linear regression between depression-PRS and each of the phenotype. Each dot represents one phenotype, and the colours indicate their according categories. The pink dashed line indicates the threshold for FDR/Bonferroni-corrected significance (as no effect survived multiple correction, thresholds for both multiple correction methods are the same).

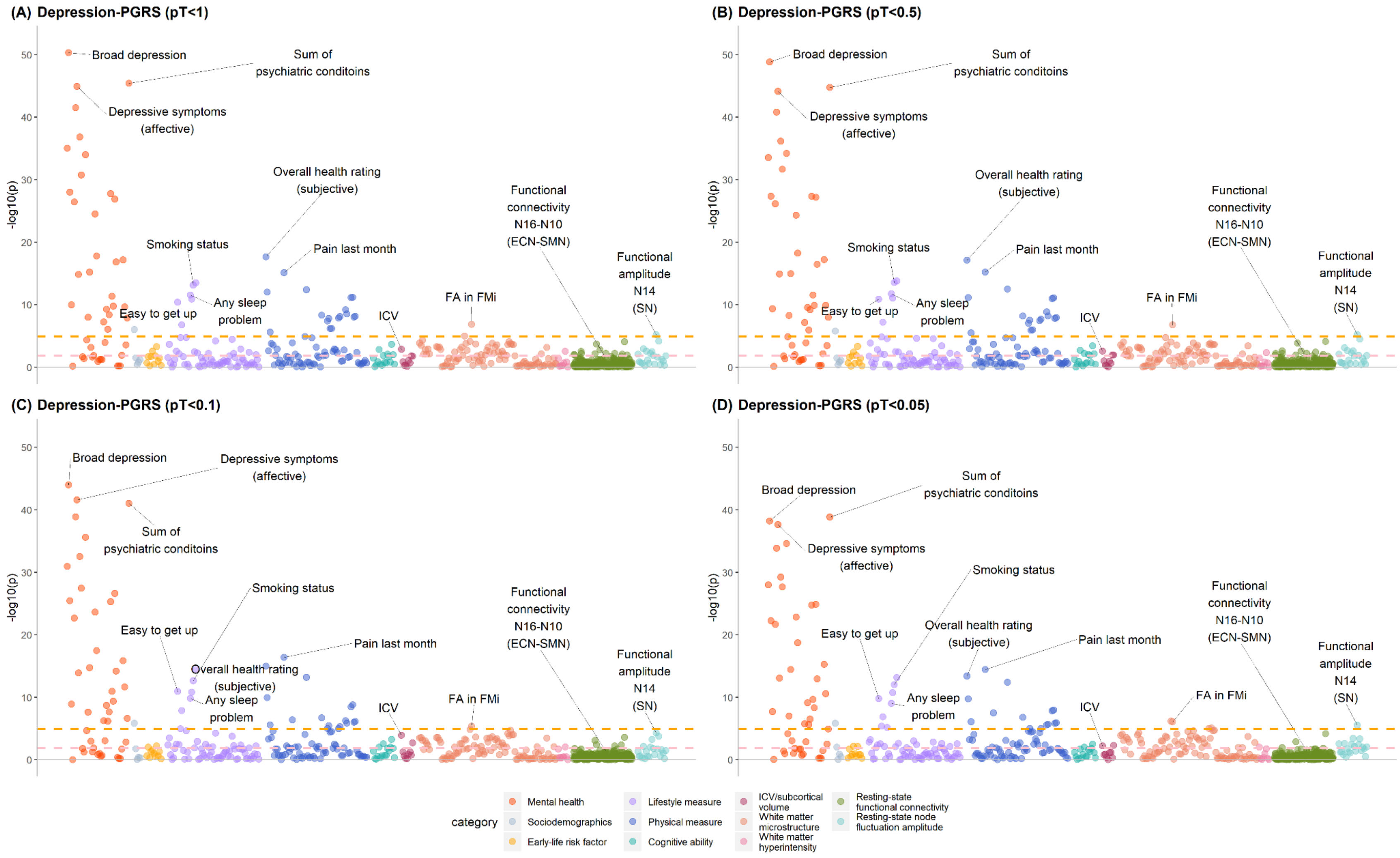
(A) Depression-PGRS (pT<1)
(B) Depression-PGRS (pT<0.5)
(C) Depression-PGRS (pT<0.1)
(D) Depression-PGRS (pT<0.05)
-log10(p)
Broad depression
Sum of psychiatric conditoins
Depressive symptoms (affective)
Overall health rating (subjective)
Smoking status
Pain last month
Easy to get up
Any sleep problem
ICV
FA in FMi
Functional connectivity N16-N10 (ECN-SMN)
Functional amplitude N14 (SN)
category
Mental health
Sociodemographics
Early-life risk factor
Lifestyle measure
Physical measure
Cognitive ability
ICV/subcortical volume
White matter microstructure
White matter hyperintensity
Resting-state functional connectivity
Resting-state node fluctuation amplitude

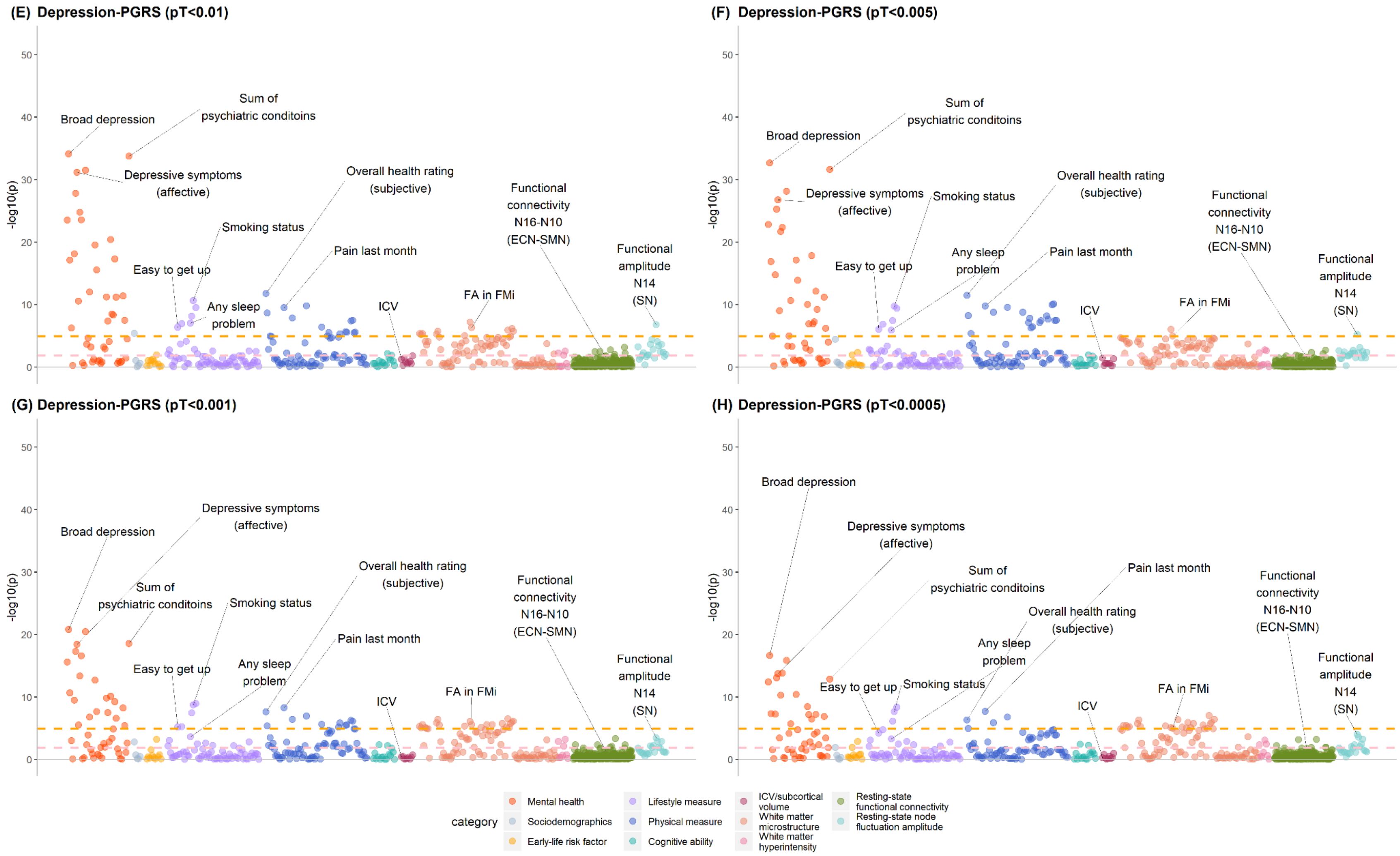

Supplementary Figure 5. Significance plots for PheWAS results on all depression-PGRS p thresholds (pT) in the total sample including both the discovery and the replication samples (N=21,888). Panels (A-D) are shown on this page and the other panels (E-H) are shown on the next page. The x axes represent phenotypes, and the y axes represent the -$\log_{10}$ of uncorrected p values of two-sided test for linear regression between depression-PRS and each of the phenotype. Each dot represents one phenotype, and the colours indicate their according categories. Top associations found in depression-PGRS at pT<1 and pT<0.01 (see the main text) are annotated in each panel. The pink dashed line indicates the threshold for FDR-corrected significance. The orange dashed line indicates the threshold for Bonferroni-corrected significance.

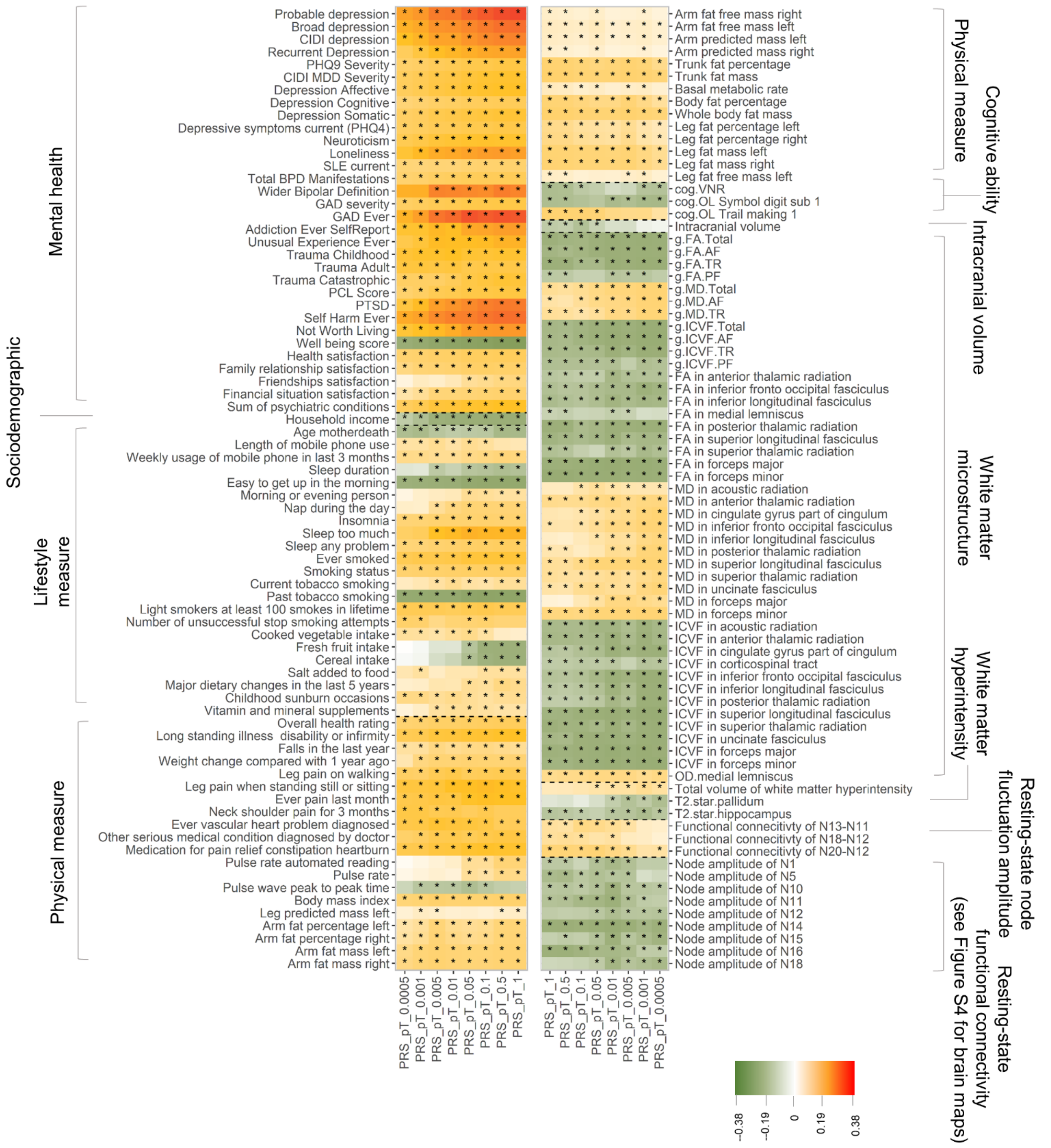

Supplementary Figure 6. Heatmap for the traits that associated with depression-PGRS at four minimum four pT in the total sample including both the discovery and the replication samples (N=21,888). Shades of cells indicate the standardised effect sizes (β) for the linear regression between depression-PRS and each phenotype. A larger effect size was shown by a darker colour. Cells with an asterisk were significant after FDR-correction.

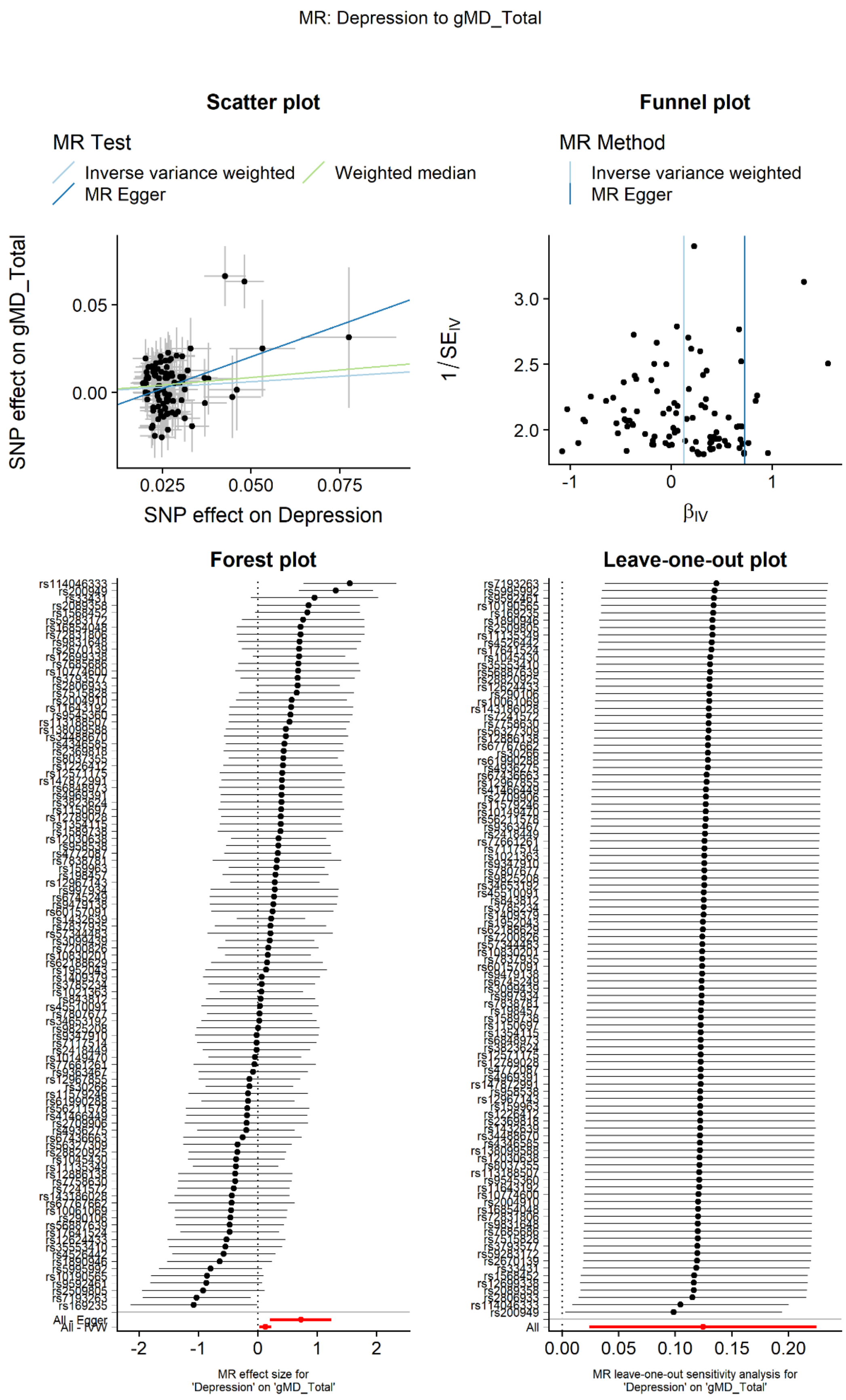

Supplementary Figure 7. Mendelian Randomisation analysis testing the causal effect of depression on gMD-Total (general variance of mean diffusivity).

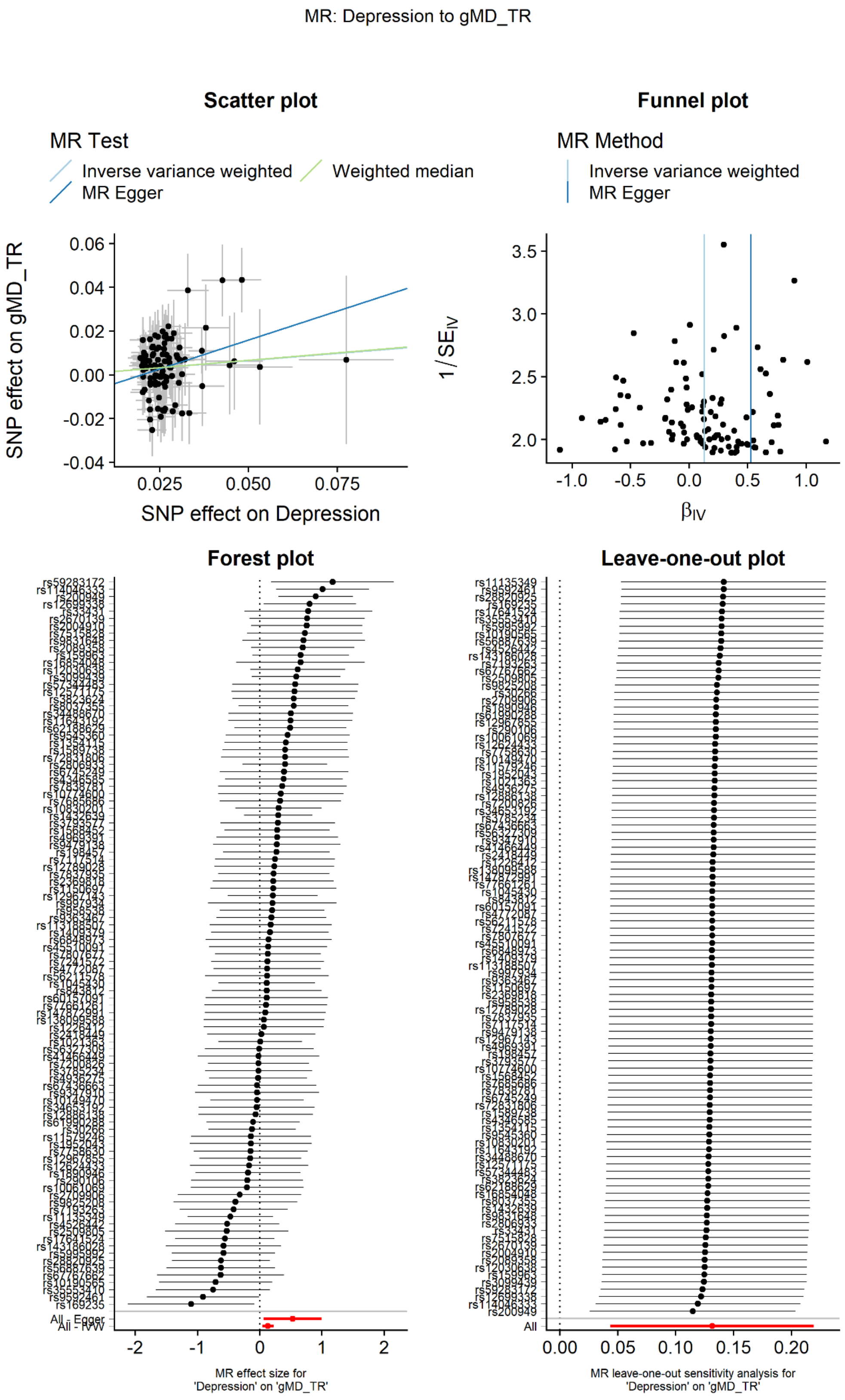

Supplementary Figure 8. Mendelian Randomisation analysis testing the causal effect of depression on gMD-TR (general variance of mean diffusivity in thalamic radiations).

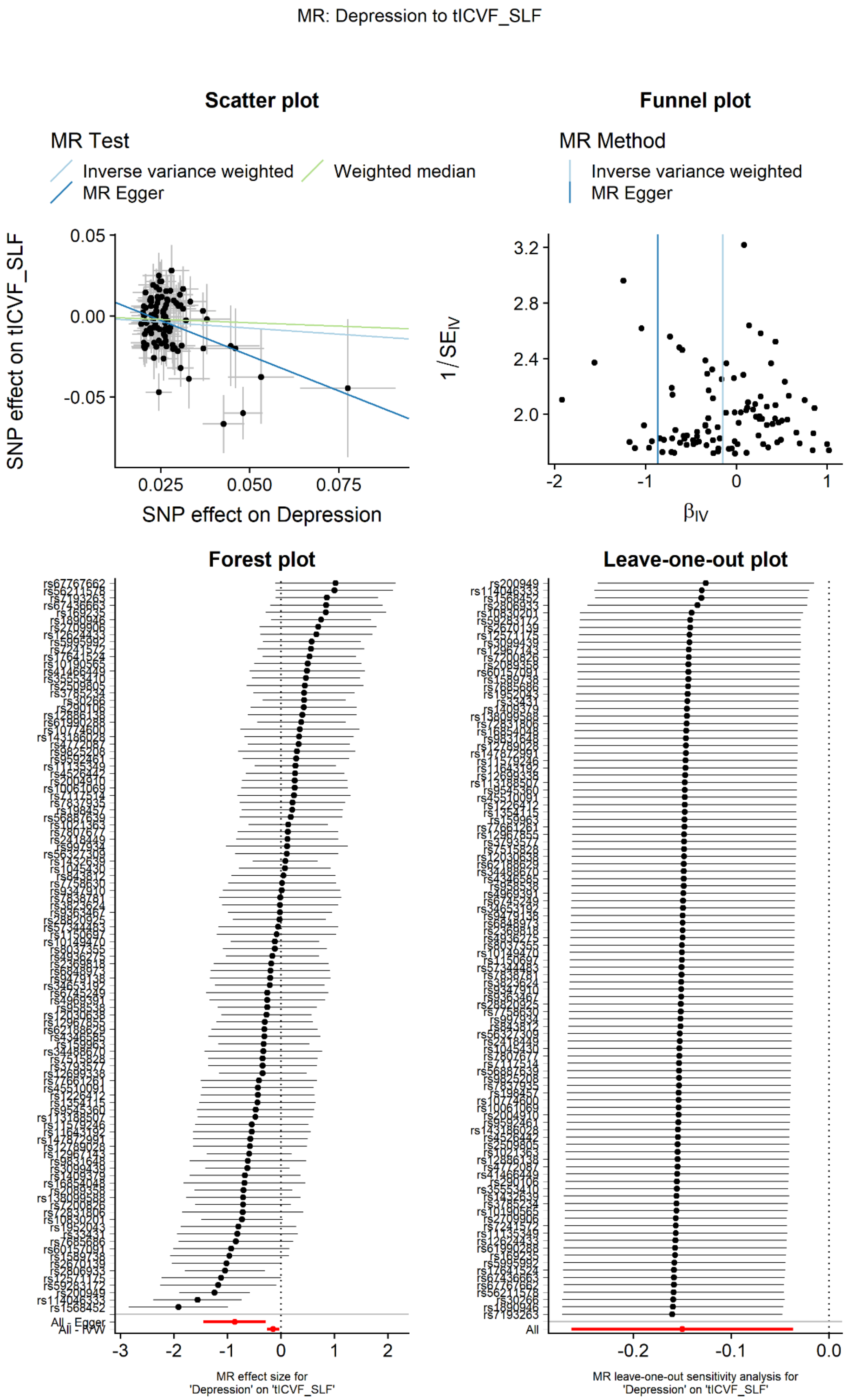

Supplementary Figure 9. Mendelian Randomisation analysis testing the causal effect of depression on intra-cellular volume fraction in superior longitudinal fasciculus.

MR: Depression to tICVF_FMa

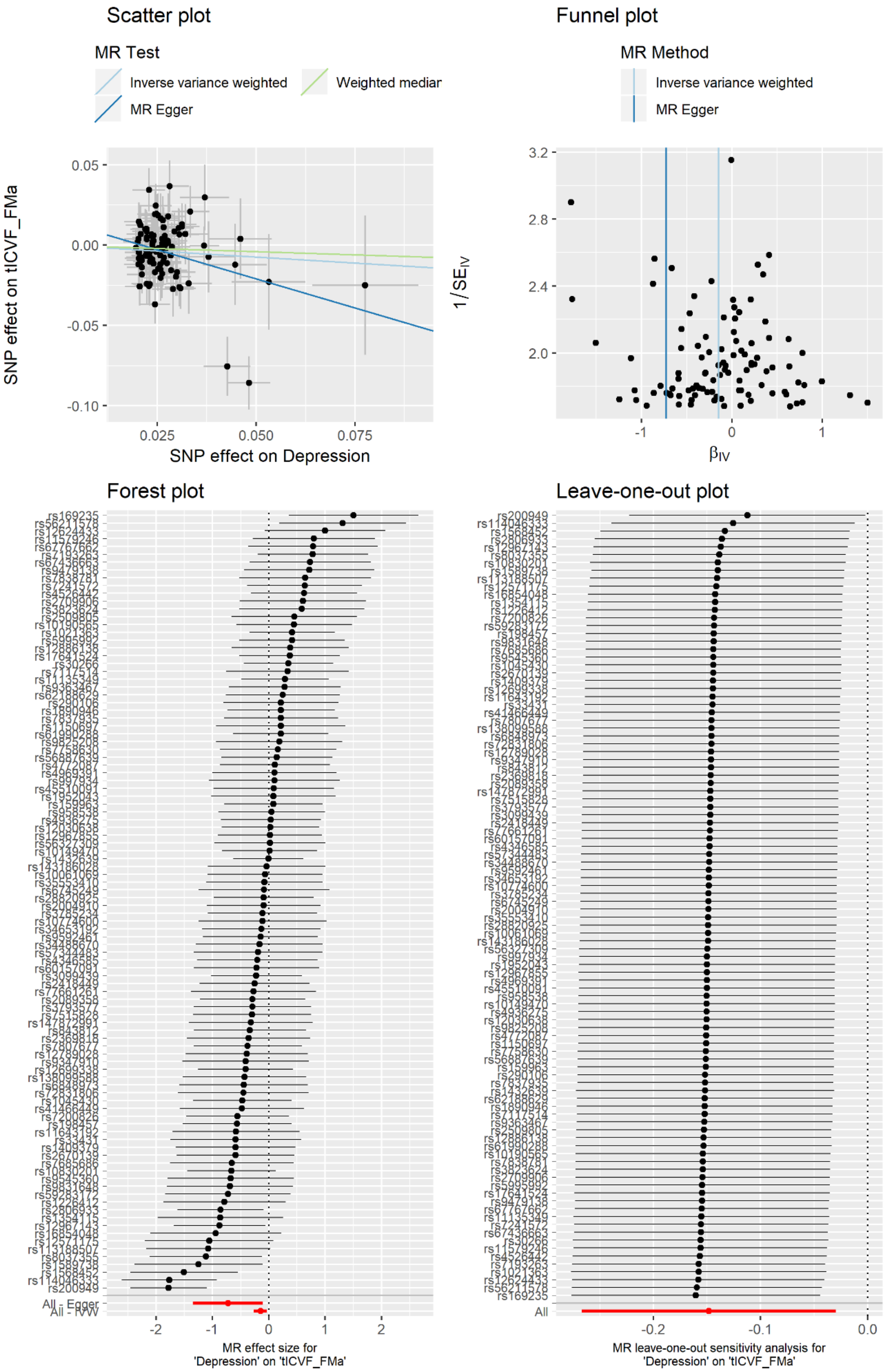

Supplementary Figure 10. Mendelian Randomisation analysis testing the causal effect of depression on intra-cellular volume fraction in forceps major.

MR: tMD_ATR to Depression

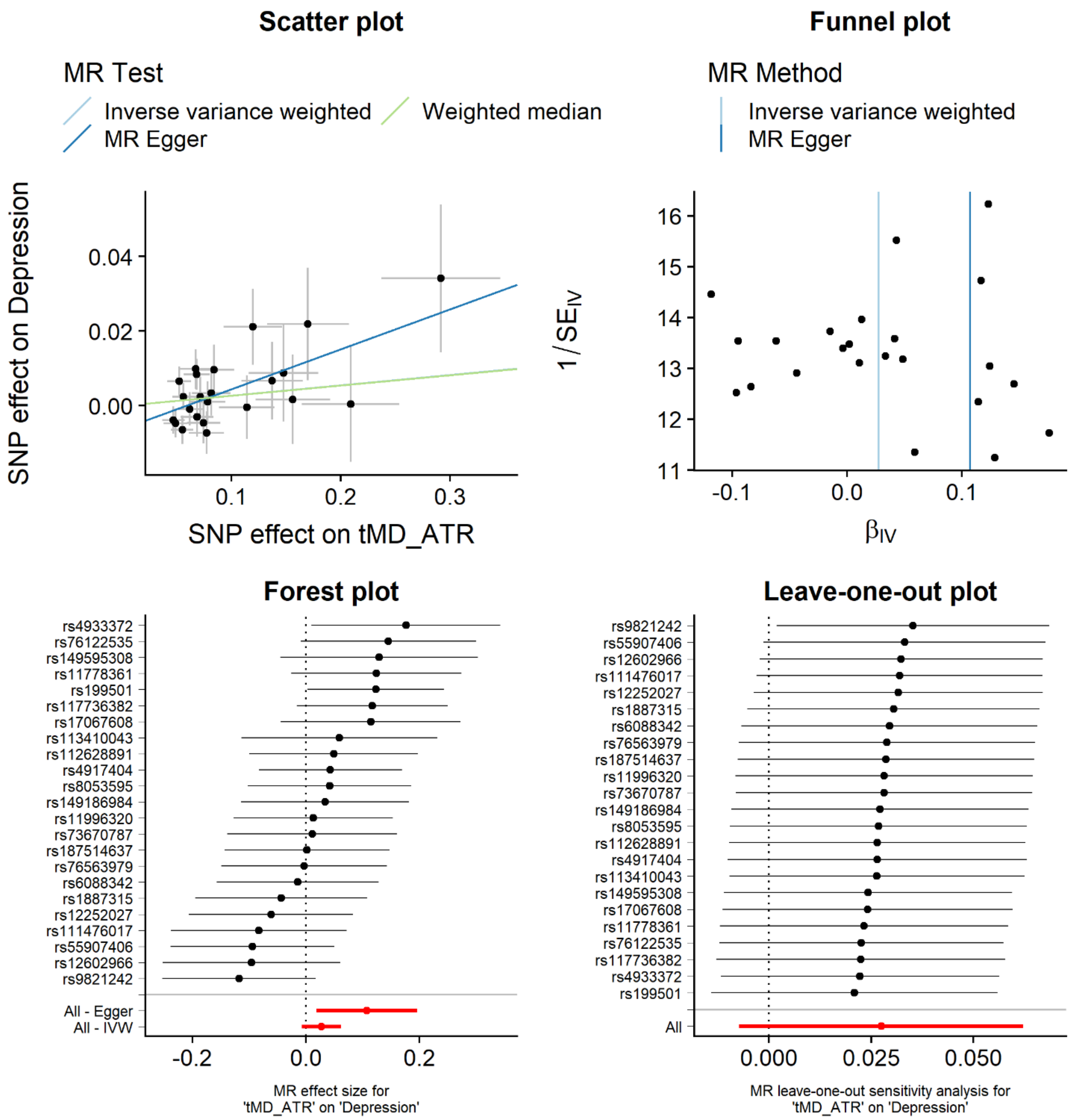

Supplementary Figure 11. Mendelian Randomisation analysis testing the causal effect of mean diffusivity in anterior thalamic radiation on depression.

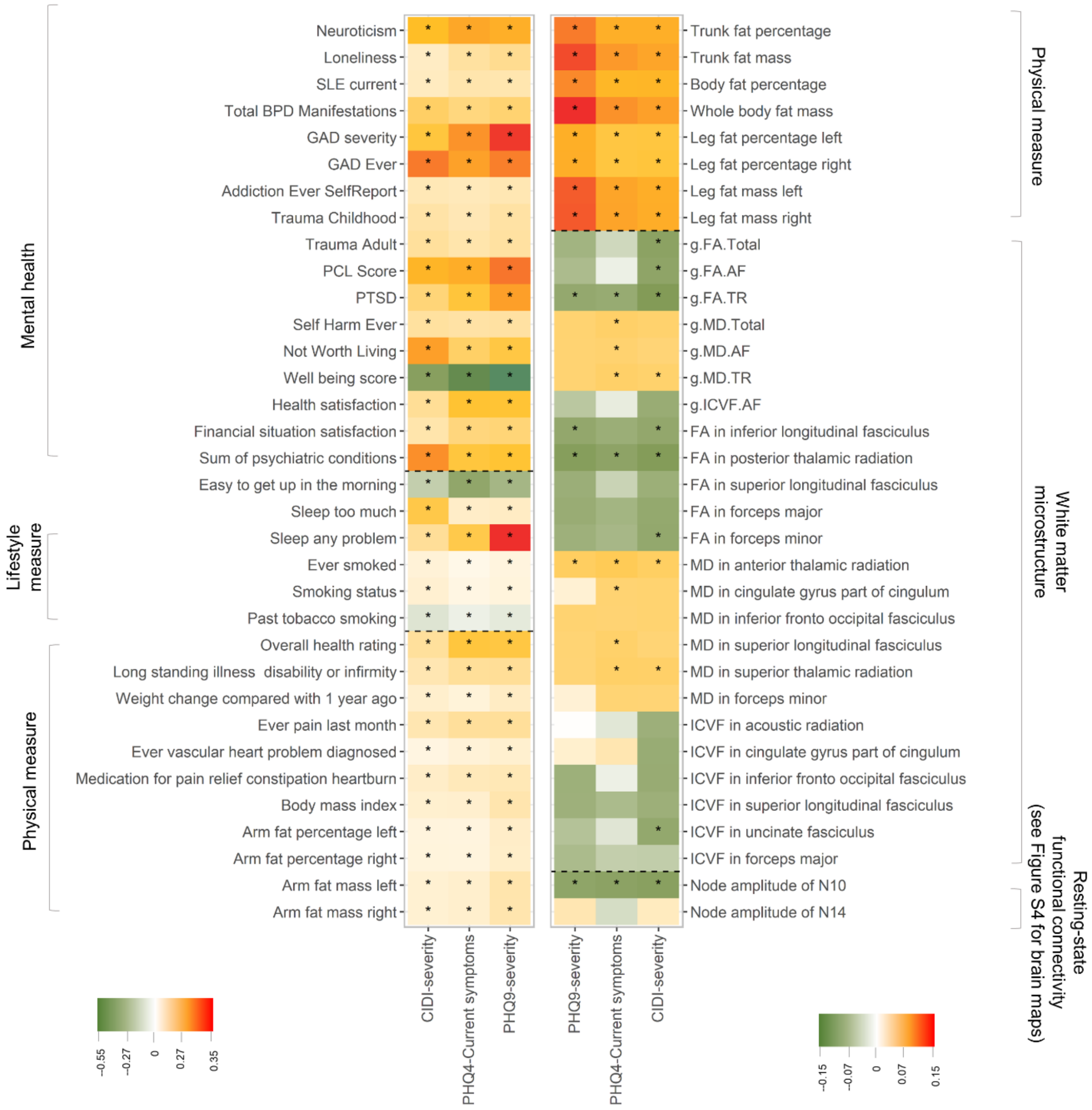

Supplementary Figure 12. Heatmap for phenotypic correlations between depressive symptoms (general depressive symptoms assessed by Composite International Diagnostic Interview-short form, Patient Health Questionnaire (PHQ)-9, and current symptoms assessed by PHQ-4).

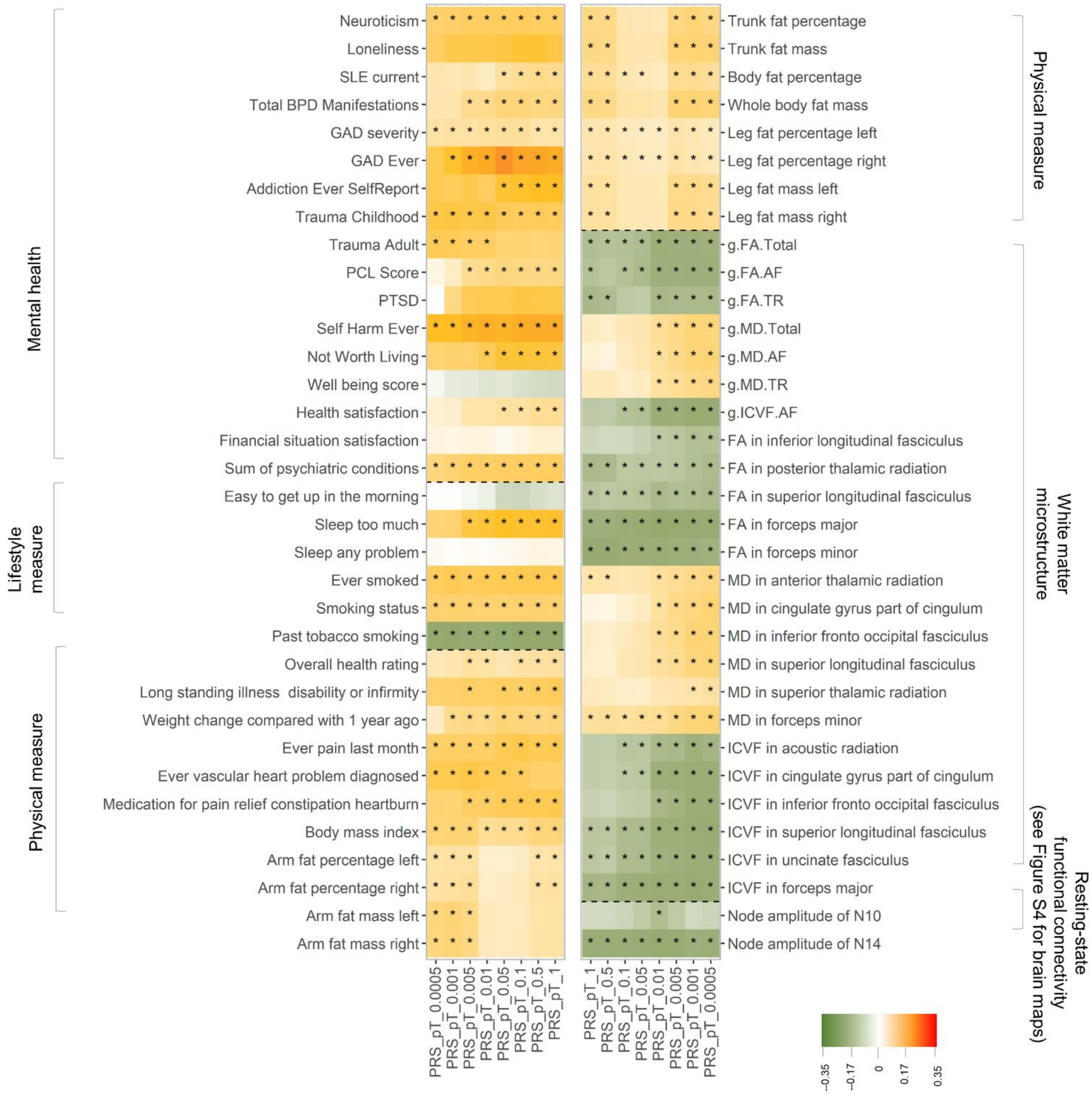

Supplementary Figure 13. Heatmap for replicated associations, correcting for depressive symptoms (assessed by Composite International Diagnostic Interview-short form, Patient Health Questionnaire (PHQ)-9, and current symptoms assessed by PHQ-4). Decreased effect sizes can be seen compared with Supplementary Figure 14.

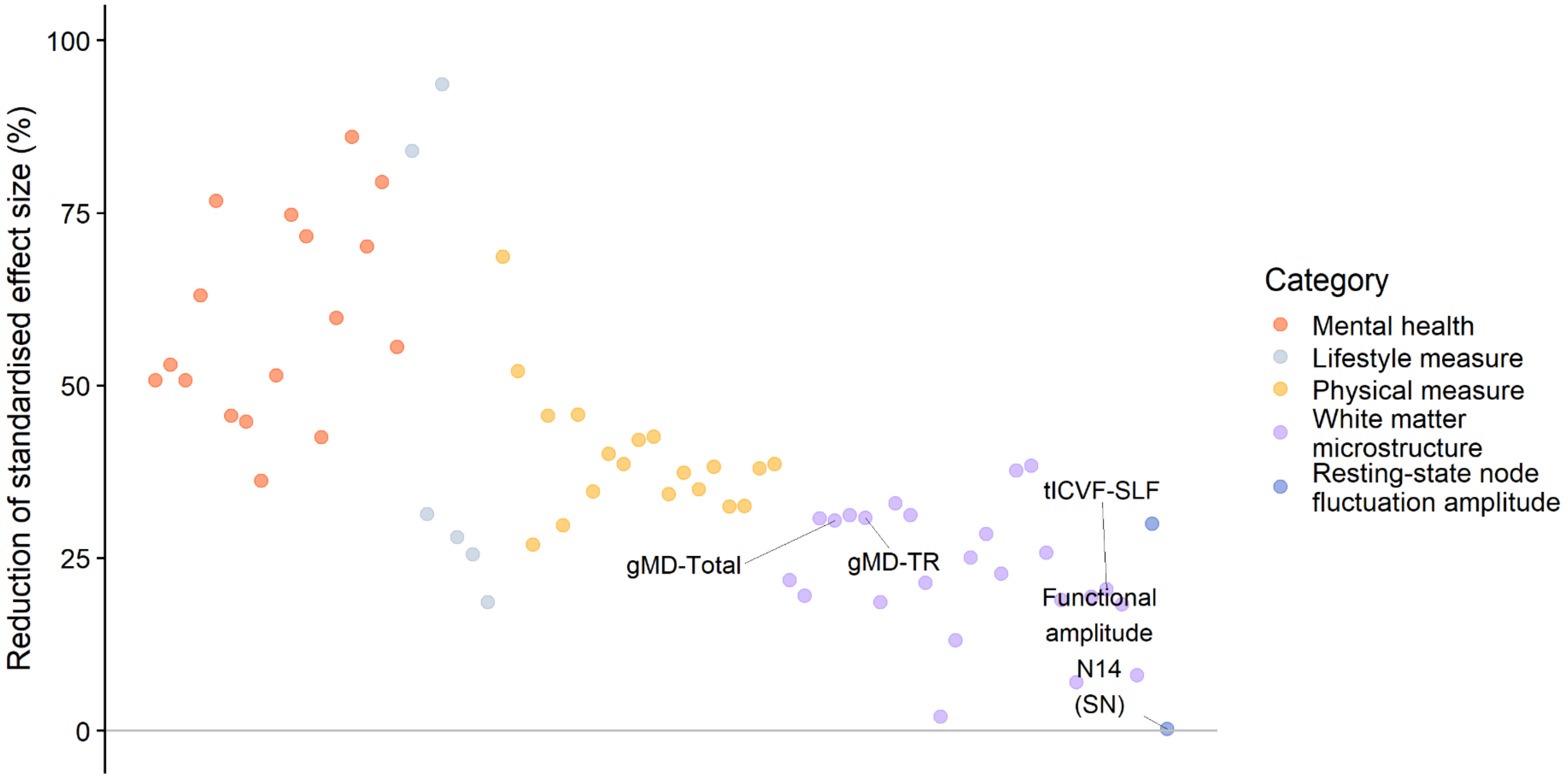

Supplementary Figure 14. Attenuated effect sizes after controlling for depressive symptoms (additional covariates consistent with Supplementary Figure 12-13). X-axis represents the replicated phenotypes. Y-axis represents the percentage of reduction in mean effect sizes across PRSs under all thresholds, after controlling for depressive symptoms. Tabulated phenotypes were the significant causal consequences for depression based on Mendelian Randomisation analysis. Other than functional amplitude of node 14, all other phenotypes showed a relatively large attenuation of effect. In the figure, gMD-Total = general variance of mean diffusivity, gMD-TR = general variance of mean diffusivity in thalamic radiations, tICVF-SLF = tract neurite density in superior longitudinal fasciculus, and SN = salience network.

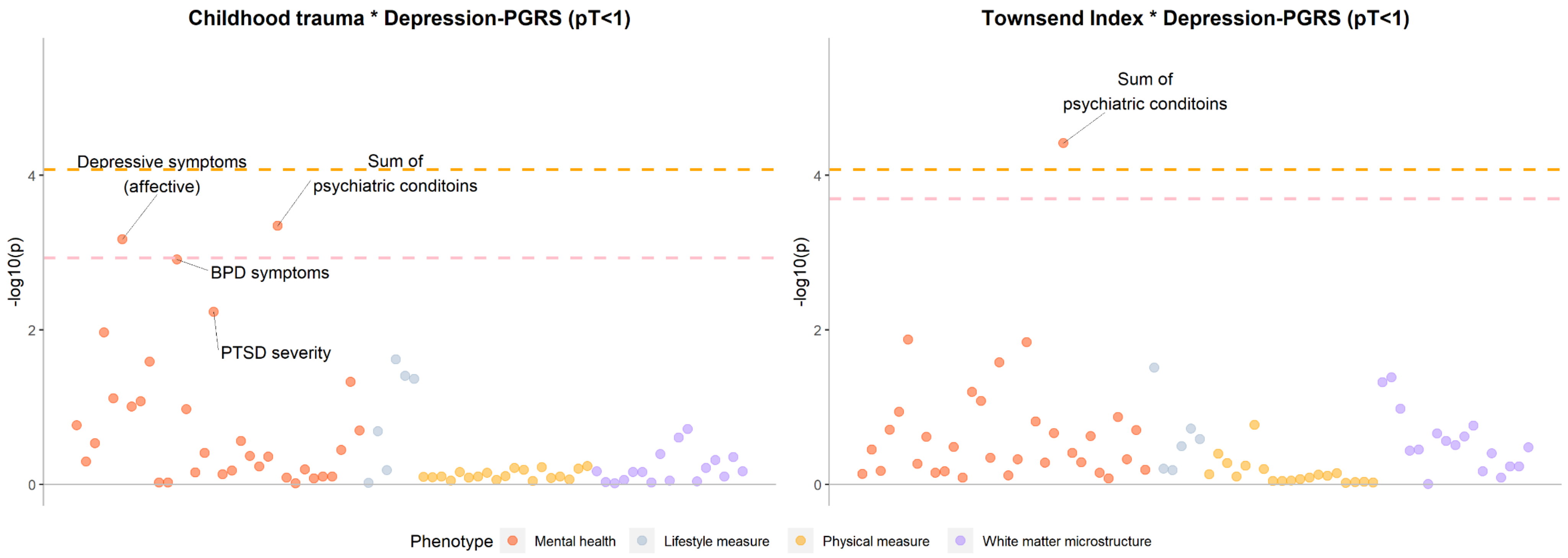

Supplementary Figure 15. Interaction between depression-PRS and variables including sociodemographic variables and early-life risk factors for depression. Dependent variables are the traits that showed significant associations with depression-PRS in at least four thresholds in both the discovery and replication samples. In the panels, each dot represents the p value for a dependent variable. The x axes represent phenotypes, and the y axes represent the -$\log_{10}$ of uncorrected p values of two-sided test for linear regression between depression-PRS and each of the phenotype. Each dot represents one phenotype, and the colours indicate their according categories. The pink dashed lines represent the FDR-corrected significance threshold (q_{FDR}=0.05), and the orange dashed lines represent the Bonferroni-corrected significance threshold.

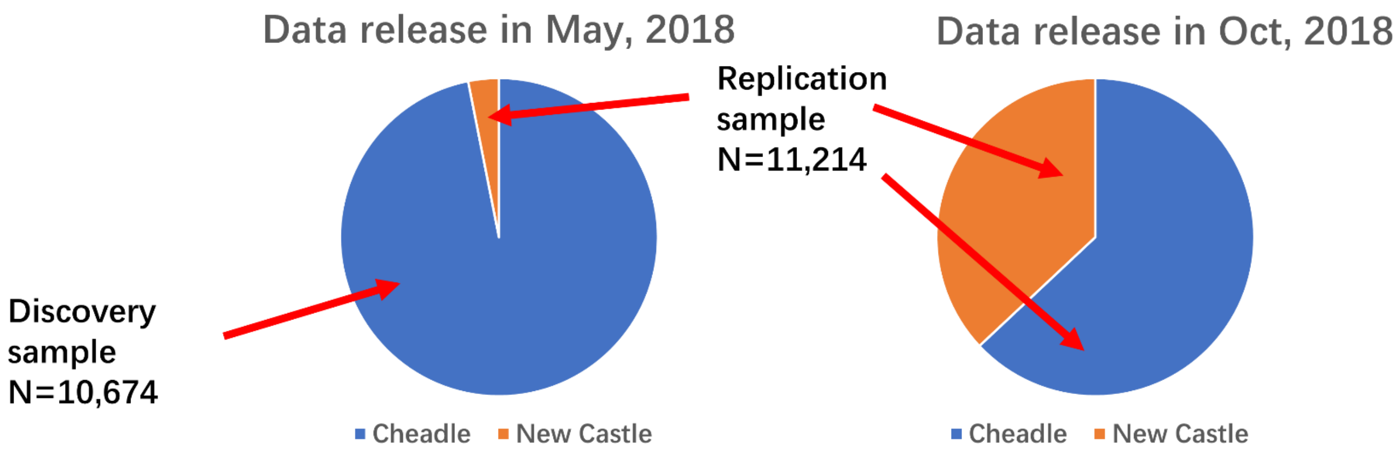

Supplementary Figure 16. Illustration of the discovery and replication samples. For the release in May 2018, 10,674 people attended the site in Cheadle, and 343 people participated in New Castle. For the other release in Oct 2018, 6,850 people attended the site in Cheadle, and 4,021 people participated in New Castle. For data collected in Cheadle, there were 17,524 people in total (49.3% were men, mean age = 63.38 years, SD of age = 7.49 years). There were 4,364 people assessed at Newcastle (47.3% were men, mean age = 64.49 years, SD of age = 7.36 years). Combining the samples from Cheadle and Newcastle, there were 21,888 participants in total (48.9% were men, mean age = 63.66 years, SD of age = 7.48 years).

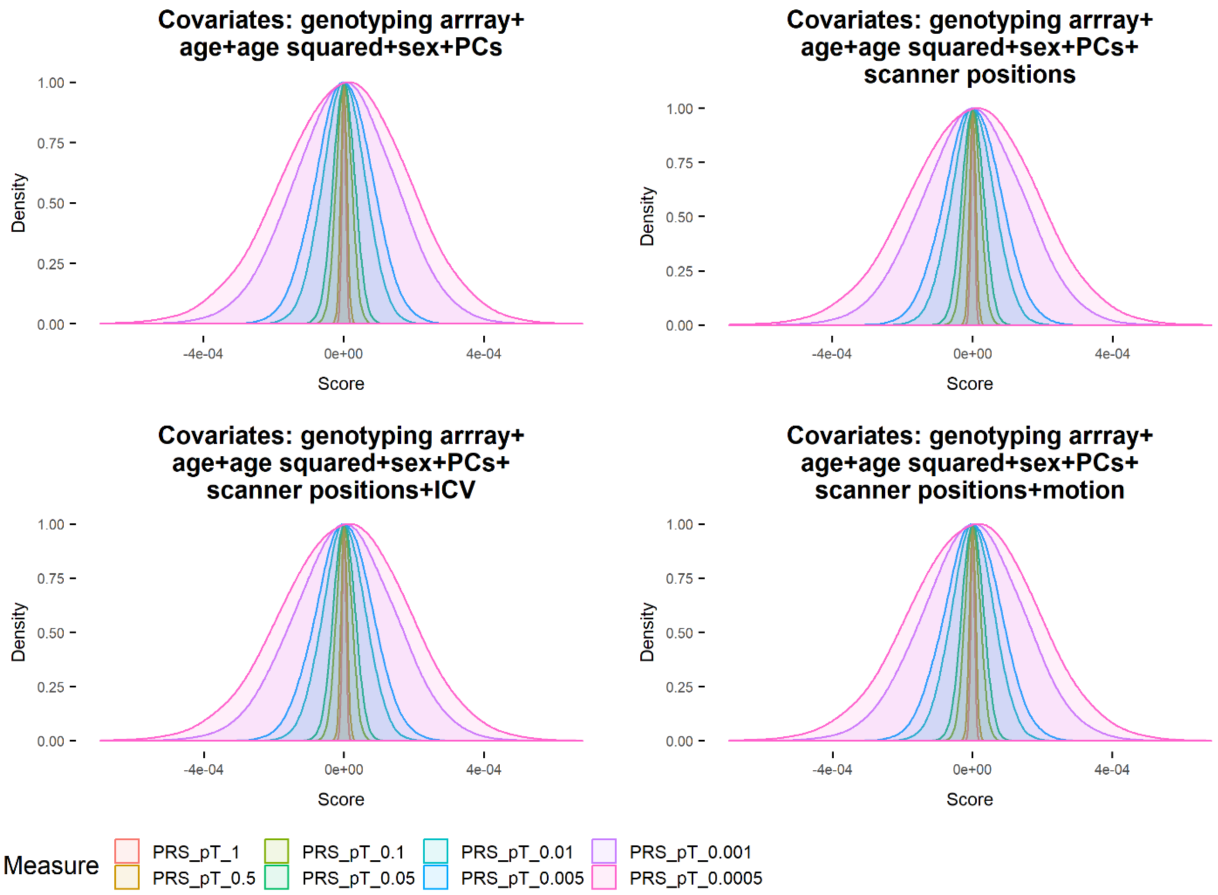

Supplementary Figure 17. Distributions of PRSs using different covariates. Plots were made using ggplot2, with an adjustment index = 2, alpha = 0.1 for illustration.

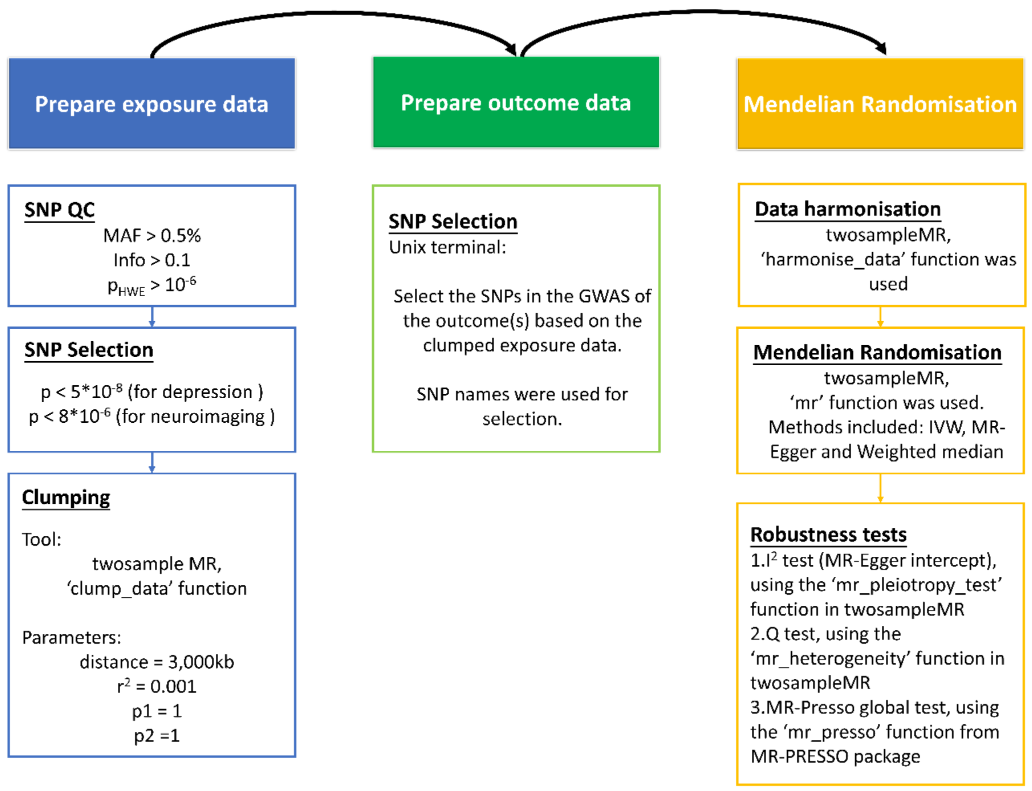

Supplementary Figure 18. Flow-chart for the procedures of Mendelian Randomisation analyses. In the chart, from left to right, the main steps are listed by sequence (the colour-filled labels). Within each major step, the procedures are listed underneath the main labels (as the boxes with colours consistent with the main labels).

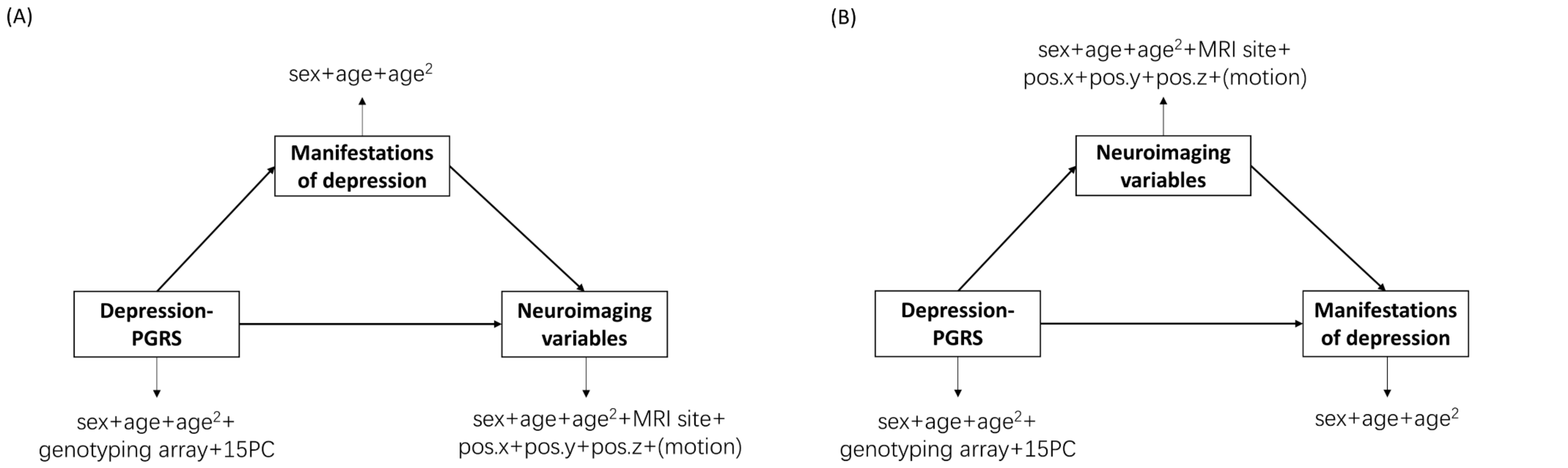

Supplementary Figure 19. Two types of mediation models. (A) Mediation model testing the mediating effect of manifestations of depression for the association between genetic risk and neuroimaging variables. (B) Mediation model testing the mediating effect of neuroimaging variables for the genetic association of depression. Neuroimaging variables were selected based on the results from Mendelian Randomisation analyses. Those that showed as causal consequences of depression were tested in model (A) and the ones showed significant causal effect to depression were tested in model (B). Variables that were considered as manifestations of depression include CIDI (Composite International Diagnostic) depression, severity of depression assessed by CIDI short form and current depressive symptoms assessed by PHQ(Patient Health Questionnaire)-4 at the imaging assessment. The results for depression-PRS is reported in the main text.

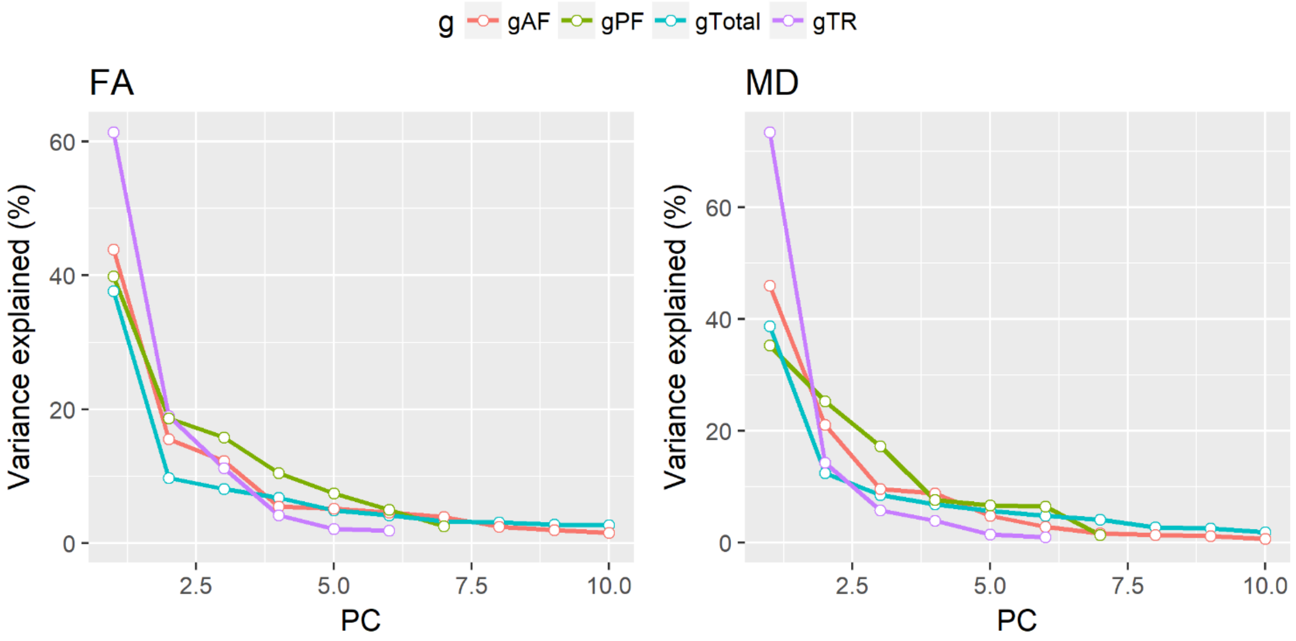

Supplementary Figure 20. Scree plots of Principal Component Analysis on fractional anisotropy (FA) and mean diffusivity (MD). The x axes represent the principal components. The y axes represent variance explained by each principal component. PCA was conducted on (i) all the tracts (gTotal), (ii) association fibres (gAF), (iii) thalamic radiations (gTR) and (iv) projection fibres (gPF).

Supplementary Table 1. Additional information for phenotypes.

Category	General description	Covariates
Mental health	Mental health questionnaires from Touchscreen's mental-health section and questions from the online follow-up section were included.	Age, age^2, sex,genotyping array, 15 genetic principal components (PCs)
Sociodemographic	Items include education, household income, ethnicity, immigration status and social deprivation.	
Early-life risk factor	Self-declared early-life risk factors. Mainly derived based on another study by Ruth et al[14]. Items include developmental factors such as birth weight and comparative weight and height at early ages. Ages of parental death were additionally included as parental factors.	
Lifestyle measures	Self-declared lifestyle questions, mainly including items relevant to sleep quality, smoking, alcohol consumption, usage of electronic devices, food and beverage intake, appearance, and social activities.	
Physical measures	This category contains data from self-declared physical conditions from the Touchscreen data modality and measured physical data from the Physical Measures modality.	
Cognitive ability	Pair matching, reaction time, and verbal-numeric reasoning tasks were conducted at the same occasion with imaging assessment. Prospective memory, pair matching, symbolic digital substitution, verbal-numeric reasoning and trail making tasks were conducted online[15]. A full list of tasks can be found in Supplementary Table 18. A variable of g score was added, deriving from the scores of the first principal component using unrotated PCA on the tasks completed at the assessment centres.	
Intracranial/subcortical volume	Measures were derived from T1 data. Eight subcortical regions were mapped and measured. A derived measure of the intracranial volume was generated by adding up the volumes for grey and white matter and ventricular cerebrospinal fluid.	Age, age^2, sex, genotyping array, 15 PCs, ICV, scanner position on the x,y and z axes
White matter hyperintensity	Using T2 flair data. Measures include a total volumetric measure of white matter hyperintensity over the whole brain and measures for each seven subcortical regions.	
White matter microstructure	Weighted-mean fractional anisotropy and mean diffusivity and three NODDI (neurite orientation dispersion and density imaging) measures of major tracts were derived for 27 major tracts (12 bilateral and 3 unilateral), mapped using probabilistic tractography. In addition to individual tracts, general variance in all tracts (gTotal) and variance in association/commissural fibres (gAF), thalamic radiations (gTR) and projection fibres (gPF) were derived using principal component analysis.	

Resting-state functional connectivity	Two-two paired, partial correlation matrix of 21 parcellated nodes generated by group-ICA was estimated and used as a measure for functional connectivity.	Age, age^2, sex, genotyping array, 15 PCs, mean motion, scanner position on the x,y and z axes
Resting-state fluctuation amplitude	Fluctuation amplitude of low-frequency signal in the 21 parcellated nodes.	

Supplementary Table 2. List of regions for the resting-state node amplitude that negatively associated with higher Depression-PGRS. The report was generated using the 'report' function in the 'xjview' package in Statistical Parametric Mapping (SPM) version 12.

Coordination of the voxel with the highest intensity in the cluster	AAL region	Cluster size	Highest intensity
-10, 54, 2	Cingulum_Ant_L (aal)	7065	-0.70989
-44, -30, 46	Postcentral_L (aal)	2781	-0.96258
44, -24, 40	Postcentral_R (aal)	1619	-0.79525
-38, -4, 16	Insula_L (aal)	963	-0.75424
28, -54, -22	Cerebelum_6_R (aal)	519	-0.63821
22, -58, -48	Cerebelum_8_R (aal)	427	-0.81925
40, -2, 16	Insula_R (aal)	407	-0.59333
30, 18, -16	Insula_R (aal)	308	-0.41634
-24, -52, -20	Cerebelum_6_L (aal)	268	-0.53087
32, 36, -10	Frontal_Inf_Orb_R (aal)	171	-0.44853
-34, 34, -12	Frontal_Inf_Orb_L (aal)	154	-0.42166
-24, -54, -50	Cerebelum_8_L (aal)	150	-0.54941
-18, 32, 38	Frontal_Sup_L (aal)	124	-0.40362
2, -64, -18	Vermis_6 (aal)	70	-0.42048
-58, 6, 36	Precentral_L (aal)	47	-0.36725

Supplementary Table 3. Genetic instruments used for the Mendelian Randomisation analysis of mean diffusivity of anterior thalamic radiation to depression. SNPs were chosen from the GWAS of mean diffusivity in anterior thalamic radiation. Beta and double-side, uncorrected p values are reported for GWAS of mean diffusivity (MD) in anterior thalamic radiation and GWAS for depression. Nearest gene(s) are reported based on information from the 'Oxford Big' website (big.stats.ox.ac.uk). Traits that were found associated with these nearest gene(s) were also reported, based on the 'GWAS Catalog' (https://www.ebi.ac.uk/gwas/). In the table, MD-ATR = MD in anterior thalamic radiation.

**SNP**	**Effect allele for MD-ATR GWAS**	**Other allele for MD-ATR GWAS**	**Frequency of effect allele for MD-ATR GWAS**	**Beta: MD-ATR GWAS**	**P-value：MD-ATR GWAS**	**Odds ratio: depression GWAS**	**P-value: depression GWAS**	**Nearest gene(s)**	**Traits associated with the nearest gene(s)**
rs17067608	G	A	0.9173046	8.4e-02 (1.9e-02)	0.0000061	9.6e-03 (6.8e-03)	0.16	QRSL1	Daytime rest measurement
rs8053595	T	C	0.0928854	8.2e-02 (1.8e-02)	0.0000033	3.4e-03 (6.0e-03)	0.57	CTRB1	Blood protein measurement, Type 1 diabetes, Type 2 diabetes
rs11996320	A	T	0.888405	7.8e-02 (1.6e-02)	0.0000012	1.0e-03 (5.6e-03)	0.86	NCALD	Neuropsychological test, Alcohol dependence, Unipolar depression, Cerebral amyloid angiopathy, FEV/FEC ratio, Systolic blood pressure
rs55907406	T	A	0.89065	7.7e-02 (1.6e-02)	0.0000016	-7.3e-03 (5.7e-03)	0.2	MYOCD	
rs12252027	T	G	0.123286	7.4e-02 (1.5e-02)	0.0000011	-4.6e-03 (5.5e-03)	0.41	HTRA1	Coronary artery disease, Laterality measurement, Lung function, Systolic blood pressure

rs149186984	G	A	0.130978	7.2e-02 (1.5e-02)	0.0000017	2.4e-03 (5.4e-03)	0.65	RNF213	Moyamoya disease
rs1887315	C	G	0.870047	6.8e-02 (1.5e-02)	0.0000048	-3.0e-03 (5.3e-03)	0.57	FYN	Schizophrenia, Aggressive behavior
rs199501	A	G	0.235379	6.8e-02 (1.2e-02)	1E-08	8.4e-03 (4.2e-03)	0.045	WNT3	Parkinson's disease, Irritability, Mood instability, Coronary artery disease
rs76122535	C	G	0.865607	6.7e-02 (1.5e-02)	0.0000057	9.8e-03 (5.3e-03)	0.063	ICA1L	Smoking status measurement, Total cholesterol measurement,
rs6088342	G	A	0.805917	6.2e-02 (1.3e-02)	0.0000014	-9.0e-04 (4.5e-03)	0.83	CHMP4B	
rs4917404	T	C	0.420334	5.6e-02 (1.0e-02)	4.1E-08	2.4e-03 (3.6e-03)	0.51	OBFC1	Telomere length
rs9821242	C	T	0.571097	5.5e-02 (1.0e-02)	8.2E-08	-6.5e-03 (3.8e-03)	0.084	/	
rs11778361	T	G	0.297186	5.2e-02 (1.1e-02)	0.0000019	6.5e-03 (4.0e-03)	0.1	DMRT2	Sleep duration, Language impairment, Frontotemporal dementia, Vital capacity
rs12602966	A	C	0.687763	4.9e-02 (1.1e-02)	0.0000069	-4.7e-03 (3.9e-03)	0.22	DCAKD,NMT1	DCAKD:Heel bone mineral density, NMT1: Systolic blood pressure, Insomnia, Pulse pressure
rs111476017	A	G	0.437521	4.7e-02 (1.0e-02)	0.0000048	-3.9e-03 (3.7e-03)	0.29	RAI14	Daytime rest, Smoking behaviour, FEV/FEC ratio,

rs117736382	A	C	0.00868342	2.9e-01 (5.4e-02)	8.8E-08	3.4e-02 (2.0e-02)	0.085	IFNGR1	Cognitive decline measurement, Cognitive impairment, Body mass index, Systolic blood pressure, Vital capacity
rs187514637	C	G	0.0132578	2.1e-01 (4.5e-02)	0.0000027	4.0e-04 (1.5e-02)	0.98	CITED2	
rs149595308	G	A	0.9798172	1.7e-01 (3.7e-02)	0.000006	2.2e-02 (1.5e-02)	0.15	AQP4	Late-onset Alzheimers disease, Serum creatinine measurement, Body mass index
rs73670787	A	G	0.0234718	1.6e-01 (3.4e-02)	0.0000046	1.7e-03 (1.2e-02)	0.89	DLGAP2	Neuroticism measurement, Depressive symptom measurement, Wellbeing measurement, Self-reported educational attainment
rs113410043	A	G	0.0279627	1.5e-01 (3.2e-02)	0.0000035	8.7e-03 (1.3e-02)	0.5	ASTN2	Hippocampal volume, Response to vaccine, Migraine disorder, Unipolar depression, Adolescent idiopathic scoliosis
rs112628891	T	C	0.0340985	1.4e-01 (2.8e-02)	0.0000011	6.7e-03 (1.0e-02)	0.52	OSER1	Granulocyte percentage of myeloid white cells, Neuropsychological test, Late-onset Alzheimers disease

rs4933372	C	T	0.9629141	1.2e-01 (2.7e-02)	0.0000077	2.1e-02 (1.0e-02)	0.039	GRID1	Asthma, Anorexia nervosa, Schizophrenia, Post-traumatic stress disorder, Body mass index
rs76563979	T	A	0.0434642	1.1e-01 (2.5e-02)	0.0000067	-4.0e-04 (8.5e-03)	0.96	PIK3C3	Body mass index

Supplementary Table 4. Summary of GWAS for neuroimaging and results for LD score regression. The sample sizes are the same with the ones for PheWAS combining both discovery and replication samples, therefore for brevity, they are not included in this table. In the table, estimated partitioned heritability using LDSC, numbers of genome-wide significant hits (>3,000kb, r^2<0.001, p<5×10^{-8}) are reported. Softwares and parameters used for GWAS can be found in the methods section in the main text. In the table, AF = association fibres, TR = thalamic radiation, FMa = forceps major, FMi = forceps minor, ILF = inferior longitudinal fasciculus, PTR = posterior thalamic radiation, SLF = superior longitudinal fasciculus, AR = acoustic radiation, IFOF = inferior fronto-occipital fasciculus, UnF = uncinate fasciculus, STR = superior thalamic radiation, Cingulate Cingulum = cingulate part of cingulum Cingulum.

**Neuroimaging phenotype**	**SNP h2**	**Lambda GC**	**Mean Chi2**	**Intercept**	**Number of genome-wide significant loci**	**Number of independent genome-wide significant SNP overlapping with depression GWAS**	**Genetic correlation with depression effect size (SE)**	**Genetic correlation with depression p FDR-corrected**
gFA-AF	0.2492 (0.0408)	1.0833	1.0896	1.0183 (0.0074)	6	4	-0.0731 (0.0432)	0.2238
gFA-Total	0.2777 (0.0418)	1.0833	1.0958	1.0161 (0.0074)	5	3	-0.0471 (0.0402)	0.2413
gFA-TR	0.2752 (0.0394)	1.0833	1.0926	1.0126 (0.0067)	5	3	-0.0576 (0.0399)	0.2238
gICVF-AF	0.3076 (0.0474)	1.0833	1.1041	1.0167 (0.0083)	14	10	-0.0825 (0.0372)	0.0264
gMD-AF	0.2511 (0.0382)	1.0649	1.0802	1.008 (0.0071)	6	4	0.0168 (0.0419)	0.6884
gMD-Total	0.2717 (0.039)	1.0618	1.083	1.0052 (0.007)	4	1	0.0356 (0.0388)	0.53805
gMD-TR	0.2354 (0.0374)	1.0557	1.0685	1.0016 (0.0065)	3	0	0.0642 (0.0404)	0.3357
Resting-state fluctuation amplitude in N10	0.1381 (0.0318)	1.0315	1.0375	0.9974 (0.0066)	7	5	-0.1098 (0.0526)	0.0369
Resting-state fluctuation amplitude in N14	0.1454 (0.0379)	1.0436	1.0473	1.0047 (0.0073)	8	7	-0.123 (0.0558)	0.0369
FA in FMa	0.1324 (0.0373)	1.0375	1.052	1.0143 (0.0066)	4	2	-0.0481 (0.0523)	0.44725
FA in FMi	0.2611 (0.0442)	1.0618	1.0847	1.0097 (0.0077)	8	5	-0.1567 (0.0405)	0.0005
FA in ILF	0.2212 (0.0386)	1.0741	1.0864	1.0229 (0.0069)	6	4	0.0123 (0.045)	0.7843
FA in PTR	0.2169 (0.0366)	1.071	1.0775	1.0144 (0.0065)	4	2	-0.0672 (0.0466)	0.2495

FA in SLF	0.2698 (0.0414)	1.0864	1.0998	1.0229 (0.0076)	4	1	-0.0556 (0.0381)	0.2495
ICVF in AR	0.2541 (0.0424)	1.0649	1.0912	1.0195 (0.0081)	8	6	-0.0199 (0.0425)	0.6392
ICVF in CingulateCingulum	0.2979 (0.0445)	1.0802	1.097	1.0119 (0.0081)	11	8	-0.1009 (0.0399)	0.0228
ICVF in FMa	0.2269 (0.0449)	1.0527	1.0784	1.0141 (0.0071)	8	6	-0.0674 (0.0418)	0.12792
ICVF in IFOF	0.2863 (0.0465)	1.0772	1.0992	1.0176 (0.0081)	12	9	-0.0664 (0.0379)	0.12015
ICVF in SLF	0.3395 (0.047)	1.0833	1.1105	1.0146 (0.0078)	13	10	-0.0965 (0.0358)	0.0228
ICVF in UnF	0.262 (0.0413)	1.0864	1.0867	1.0111 (0.008)	5	4	-0.0999 (0.0392)	0.0228
MD in ATR	0.2423 (0.0343)	1.0496	1.0641	0.9951 (0.0066)	4	1	0.1062 (0.0388)	0.0122
MD in CingulateCingulum	0.2703 (0.0363)	1.0588	1.0737	0.9959 (0.0071)	5	0	0.1054 (0.0381)	0.0122
MD in FMi	0.2325 (0.039)	1.0557	1.0642	0.9975 (0.0069)	6	3	0.1189 (0.043)	0.0122
MD in IFOF	0.2767 (0.0393)	1.0618	1.0821	1.0032 (0.0072)	5	3	0.0323 (0.0376)	0.3894
MD in SLF	0.2939 (0.0438)	1.0679	1.0926	1.009 (0.0072)	6	3	0.0744 (0.0375)	0.0714
MD in STR	0.2048 (0.0364)	1.0496	1.0611	1.0033 (0.0061)	2	1	0.0777 (0.0441)	0.09372

Supplementary Table 5. Demographic information for discovery, replication and total samples.

		Discovery		Replication		Total	
Age		Mean=62.75 SD=7.44		Mean=64.41 SD=7.42		Mean=63.60 SD=7.48	
		**N**	**ratio**	**N**	**ratio**	**N**	**ratio**
**Sex (N and % for males)**		5164	48.38%	5544	49.44%	10708	48.92%
**Education**	College degree	530	5.33%	294	5.65%	824	5.44%
	Non-college degree	9411	94.67%	4905	94.35%	14316	94.56%
**Household income**	<18,000	1269	13.14%	619	12.19%	1888	12.82%
	18,000 to 30,999	2716	28.13%	1467	28.90%	4183	28.39%
	31,000 to 51,999	2935	30.40%	1540	30.33%	4475	30.38%
	52,000 to 100,000	2133	22.09%	1142	22.49%	3275	22.23%
	>100,000	602	6.24%	309	6.09%	911	6.18%
**Social deprivation (Townsend Index)**	Most deprived	727	9.01%	353	9.22%	1080	9.08%
	Middle	2348	29.10%	1067	27.86%	3415	28.70%
	Least deprived	4995	61.90%	2410	62.92%	7405	62.23%
**Current depressive symptoms (PHQ4)**		Mean=5.13 SD=1.79		Mean=5.17 SD=1.81		Mean=5.14 SD=1.80	
**MDD probable**	Case	1941	18.18%	891	7.95%	2832	12.94%
	Control	2562	24.00%	628	5.60%	3190	14.57%
**MDD broad**	Case	3218	30.15%	1541	13.74%	4759	21.74%
	Control	4599	43.09%	2200	19.62%	6799	31.06%
**MDD CIDI**	Case	1918	17.97%	905	8.07%	2823	12.90%
	Control	4573	42.84%	2136	19.05%	6709	30.65%
**Use of antidepressants (self-declared)**	Case	2374	22.24%	2312	20.62%	4686	21.41%
	Control	8300	77.76%	8902	79.38%	17202	78.59%

Supplementary Table 6. Loadings of each tract on gTotal, gAF, gTR and gPF in fractional anisotropy (FA) and mean diffusivity (MD) (see Supplementary Figure 20). The principal component analysis was conducted on the maximum sample size including both discovery and replication samples.

Tract	FA				MD			
	g.Total	gAF	gTR	gPF	g.Total	gAF	gTR	gPF
Parahippocampal part of cingulum	0.361	0.379	--	--	0.472	0.61	--	--
Parahippocampal part of cingulum	0.394	0.421	--	--	0.455	0.588	--	--
Forceps major	0.547	0.552	--	--	0.488	0.518	--	--
Cingulate gyrus part of cingulum	0.558	0.635	--	--	0.609	0.576	--	--
Cingulate gyrus part of cingulum	0.582	0.658	--	--	0.614	0.59	--	--
Uncinate fasciculus	0.649	0.666	--	--	0.665	0.7	--	--
Uncinate fasciculus	0.682	0.697	--	--	0.734	0.75	--	--
Superior longitudinal fasciculus	0.81	0.796	--	--	0.859	0.795	--	--
Superior longitudinal fasciculus	0.824	0.8	--	--	0.847	0.777	--	--
Forceps minor	0.803	0.804	--	--	0.716	0.655	--	--
Inferior longitudinal fasciculus	0.822	0.814	--	--	0.862	0.837	--	--
Inferior longitudinal fasciculus	0.85	0.827	--	--	0.893	0.85	--	--
Inferior fronto-occipital fasciculus	0.827	0.83	--	--	0.871	0.836	--	--
Inferior fronto-occipital fasciculus	0.853	0.842	--	--	0.89	0.849	--	--
Superior thalamic radiation	0.654	--	0.73	--	0.773	--	0.844	--
Superior thalamic radiation	0.65	--	0.736	--	0.743	--	0.82	--
Posterior thalamic radiation	0.676	--	0.785	--	0.756	--	0.863	--
Posterior thalamic radiation	0.775	--	0.808	--	0.82	--	0.851	--
Anterior thalamic radiation	0.693	--	0.813	--	0.775	--	0.88	--
Anterior thalamic radiation	0.773	--	0.817	--	0.811	--	0.847	--
Medial lemniscus	0.262	--	--	0.501	0.253	--	--	0.329
Middle cerebellar peduncle	0.274	--	--	0.52	0.223	--	--	0.31
Medial lemniscus	0.365	--	--	0.539	0.351	--	--	0.933
Acoustic radiation	0.608	--	--	0.577	0.486	--	--	0.307
Acoustic radiation	0.617	--	--	0.631	0.504	--	--	0.275
Corticospinal tract	0.576	--	--	0.802	0.579	--	--	0.283
Corticospinal tract	0.581	--	--	0.821	0.58	--	--	0.296

Supplementary Table 7. Results sensitivity analysis for MR (depression->neuroimaging variables). Genetic variants in low to high linkage disequilibrium with genome-wide significant SNPs of neuroticism GWAS were removed. In the table, gMD-Total=global variance of mean diffusivity, gMD-TR=variance in thalamic radiations, rsamp-N14=resting-state fluctuation amplitude in node14, tICVF-FMa=tract intra-cellular volume fraction in forceps major, tICVF-SLF=tract intra-cellular volume fraction in superior longitudinal fasciculus, IVW=inverse-variance weighted and MR-Presso outlier-corrected=MR-Presso results after removing outlying genetic instruments.

Exposure	Outcome	MR method	Nsnps	Beta	SE	p for MR	p FDR-corrected for MR	Egger intercept	SE Megger intercept	p FDR-corrected Egger intercept	Q	Q df	p FDR-corrected Q	p FDR-corrected MR-Presso
Depression	gMD-Total	IVW	96	0.135	0.052	9.43e-03	9.43e-03	-1.54e-02	7e-03	5.77e-02	112.547	95	2.11e-01	3.13e-01
Depression	gMD-Total	MR Egger	96	0.719	0.268	8.62e-03	1.72e-02	-1.54e-02	7e-03	5.77e-02	112.547	95	2.11e-01	3.13e-01
		Weighted												
Depression	gMD-Total	median	96	0.172	0.071	1.49e-02	2.98e-02	-1.54e-02	7e-03	5.77e-02	112.547	95	2.11e-01	3.13e-01
Depression	gMD-TR	IVW	96	0.143	0.046	1.77e-03	3.53e-03	-9.89e-03	6e-03	1.15e-01	87.758	95	6.88e-01	7.58e-01
Depression	gMD-TR	MR Egger	96	0.519	0.241	3.36e-02	3.36e-02	-9.89e-03	6e-03	1.15e-01	87.758	95	6.88e-01	7.58e-01
		Weighted												
Depression	gMD-TR	median	96	0.136	0.067	4.16e-02	4.16e-02	-9.89e-03	6e-03	1.15e-01	87.758	95	6.88e-01	7.58e-01
Depression	rsamp-N14	IVW	96	-0.152	0.05	2.28e-03	2.28e-03	7.87e-03	7e-03	2.48e-01	74.489	95	9.41e-01	9.66e-01
Depression	rsamp-N14	MR Egger	96	-0.451	0.262	8.86e-02	8.86e-02	7.87e-03	7e-03	2.48e-01	74.489	95	9.41e-01	9.66e-01
		Weighted												
Depression	rsamp-N14	median	96	-0.194	0.073	8.08e-03	8.08e-03	7.87e-03	7e-03	2.48e-01	74.489	95	9.41e-01	9.66e-01
Depression	tICVF-FMa	IVW	96	-0.183	0.061	2.53e-03	3.05e-03	1.67e-02	8e-03	4.22e-02	132.129	95	9.64e-03	1.23e-02
Depression	tICVF-FMa	MR Egger	96	-0.818	0.314	1.07e-02	1.07e-02	1.67e-02	8e-03	4.22e-02	132.129	95	9.64e-03	1.23e-02

Depression	tICVF-FMa	MR-Presso_outlier-corrected	94	-0.106	0.051	3.95e-02	3.95e-02	1.67e-02	8e-03	4.22e-02	132.129	95	9.64e-03	1.23e-02
Depression	tICVF-FMa	Weighted median	96	-0.106	0.075	1.56e-01	1.56e-01	1.67e-02	8e-03	4.22e-02	132.129	95	9.64e-03	1.23e-02
Depression	tICVF-SLF	IVW	96	-0.175	0.059	3.05e-03	3.05e-03	2.09e-02	8e-03	1.69e-02	130.203	95	9.64e-03	1.23e-02
Depression	tICVF-SLF	MR Egger	96	-0.969	0.301	1.75e-03	3.50e-03	2.09e-02	8e-03	1.69e-02	130.203	95	9.64e-03	1.23e-02
Depression	tICVF-SLF	MR-Presso_outlier-corrected	94	-0.124	0.052	1.86e-02	3.73e-02	2.09e-02	8e-03	1.69e-02	130.203	95	9.64e-03	1.23e-02
Depression	tICVF-SLF	Weighted median	96	-0.106	0.074	1.52e-01	1.56e-01	2.09e-02	8e-03	1.69e-02	130.203	95	9.64e-03	1.23e-02

Supplementary References

1. Bycroft, C. *et al.* The UK Biobank resource with deep phenotyping and genomic data. *Nature* **562**, 203–209 (2018).
2. Willer, C. J., Li, Y., Abecasis, G. R. & Overall, P. METAL: fast and efficient meta-analysis of genomewide association scans. *Bioinformatics* **26**, 2190–2191 (2010).
3. Warren, H. R. *et al.* Genome-wide association analysis identifies novel blood pressure loci and offers biological insights into cardiovascular risk. *Nat. Genet.* **49**, 403–415 (2017).
4. Manichaikul, A. *et al.* Robust relationship inference in genome-wide association studies. *Bioinformatics* **26**, 2867–2873 (2010).
5. Mori, S. *et al.* Imaging cortical association tracts in the human brain using diffusion-tensor-based axonal tracking. *Magn. Reson. Med.* **47**, 215–223 (2002).
6. Wakana, S., Jiang, H., Nagae-Poetscher, L. M., van Zijl, P. C. M. & Mori, S. Fiber tract-based atlas of human white matter anatomy. *Radiology* **230**, 77–87 (2004).
7. Miller, K. L. *et al.* Multimodal population brain imaging in the UK Biobank prospective epidemiological study. *Nat. Neurosci.* **19**, 1523–1536 (2016).
8. Alfaro-Almagro, F. *et al.* Image processing and Quality Control for the first 10,000 brain imaging datasets from UK Biobank. *Neuroimage* **166**, 400–424 (2018).
9. Bijsterbosch, J. *et al.* Investigations into within- and between-subject resting-state amplitude variations. *Neuroimage* **159**, 57–69 (2017).
10. Luciano, M. *et al.* Association analysis in over 329,000 individuals identifies 116 independent variants influencing neuroticism. *Nat. Genet.* **50**, 6–11 (2018).
11. Van Den Berg, S. M. *et al.* Harmonization of neuroticism and extraversion phenotypes across inventories and cohorts in the Genetics of Personality Consortium: An application of item response theory. *Behav. Genet.* **44**, 295–313 (2014).
12. Okbay, A. *et al.* Genetic variants associated with subjective well-being, depressive symptoms, and neuroticism identified through genome-wide analyses. *Nat. Genet.* **48**, 624–633 (2016).
13. Zheng, J. *et al.* LD Hub: a centralized database and web interface to perform LD score regression that maximizes the potential of summary level GWAS data for SNP heritability and genetic correlation analysis. *Bioinformatics* **33**, 272–279 (2017).

14. Ruth, K. S. *et al.* Events in Early Life are Associated with Female Reproductive Ageing: A UK Biobank Study. *Sci. Rep.* (2016). doi:10.1038/srep24710

15. Fawns-Ritchie, C. & Deary, I. J. Reliability and validity of the UK Biobank cognitive tests. *Preprint at:* doi:10.30806/fs.24.3.201909.5 (2019).
