# Supplementary material for: A phenome-wide association and Mendelian Randomisation study of polygenic risk for depression in UK Biobank
Source: Nat Commun. 2020 May 8;11:2301. doi: 10.1038/s41467-020-16022-0 (PMC7210889; doi:10.1038/s41467-020-16022-0)
Supplement: Supplementary file 18 — Description of Additional Supplementary Files [file 41467_2020_16022_MOESM18_ESM.pdf]

Title: Supplementary Data 1.
Description: Descriptions of phenotypes included in the PheWAS. In this file, all phenotypes included in the analyses were listed with their names and the according field names in the UK Biobank data showcase.

Title: Supplementary Data 2.
Description: Results for PheWAS conducted on the discovery. Where the dependent variable was binary, logistic regression and linear regression models were both conducted and therefore both Beta (standardised regression coefficient for linear regression) and Log odds ratio were reported. Two-sided, uncorrected p-values and FDR-corrected p-values are both reported for the linear regression associations between depression-PRS and each phenotype.

Title: Supplementary Data 3.
Description: Results for PheWAS conducted on the replication. Where the dependent variable was binary, logistic regression and linear regression models were both conducted and therefore both Beta (standardised regression coefficient for linear regression) and Log odds ratio were reported. Only traits that were significantly associated with depression-PGRS at a minimum of four p thresholds in the discovery dataset were tested and reported. Two-sided, uncorrected p-values and FDR-corrected p-values are both reported for the associations between depression-PRS and each phenotype.

Title: Supplementary Data 4.
Description: Site*PRS interaction on the total sample (N=21,888). Where the dependent variable was binary, logistic regression and linear regression models were both conducted and therefore both Beta (standardised regression coefficient for linear regression) and Log odds ratio were reported. Two-sided, uncorrected p-values and FDR-corrected p-values are both reported for the linear regression associations between depression-PRS and each phenotype.

Title: Supplementary Data 5.
Description: Results for PheWAS conducted on the total sample (N=21,888). Where the dependent variable was binary, logistic regression and linear regression models were both conducted and therefore both Beta (standardised regression coefficient for linear regression) and Log odds ratio were reported. Two-sided, uncorrected p-values and FDR-corrected p-values are both reported for the linear regression associations between depression-PRS and each phenotype.

Title: Supplementary Data 6.
Description: Results for Mendelian Randomisation. Double-sided uncorrected and FDR-corrected p values for the MR analyses using inverse-variance weighted (indicated as IVW in the Data file), MR-Egger and weighted median are reported, and the p values for tests for robustness were FDR-corrected. In the Data file, AF = association fibres, TR = thalamic radiation, FMa = forceps major, FMi = forceps minor, ILF = inferior longitudinal fasciculus, PTR = posterior thalamic radiation, SLF = superior longitudinal fasciculus, AR = acoustic radiation, IFOF = inferior fronto-occipital fasciculus, UnF = uncinate fasciculus, STR = superior thalamic radiation, Cingulate Cingulum = cingulate part of cingulum Cingulum.

Title: Supplementary Data 7.

Description: Genetic instruments used for the Mendelian Randomisation analysis of depression to neuroimaging variables. SNPs were chosen from depression GWAS. In the Data file, Beta value (standardised regression coefficient) for the GWAS of depression and relevant neuroimaging traits are reported. Along with the Beta values, standard deviations are reported in the brackets. Double-sided, uncorrected p-values are reported for the GWAS. For abbreviations, AF = association fibres, TR = thalamic radiation, FMa = forceps major, FMi = forceps minor, ILF = inferior longitudinal fasciculus, PTR = posterior thalamic radiation, SLF = superior longitudinal fasciculus, AR = acoustic radiation, IFOF = inferior fronto-occipital fasciculus, UnF = uncinate fasciculus, STR = superior thalamic radiation, Cingulate Cingulum = cingulate part of cingulum Cingulum.

Title: Supplementary Data 8.

Description: Model statistics for SEM models. Predictors were depression-PGRS, mediators were neuroimaging traits/manifestations of depression, and dependent variables were manifestations of depression/neuroimaging traits. In the Supplementary Data file, Beta values (standardised regression coefficients for the mediational paths), according standard error, z-scores, double-sided uncorrected p-values and FDR-corrected p-values are reported. Change of direct effect was calculated by dividing the beta values for direct path after adding the mediational path by the beta values before adding the mediational path. The fitness of the models are assessed by Comparative Fit Index (CFI in the Data file), Tucker-Lewis Index (TLI in the Data file), Root Mean Square Error of Approximation (RMSEA in the Data file) and the double-sided, uncorrected p-values for comparing RMSEA values against point value of 0.05.

Title: Supplementary Data 9.

Description: Results for gene by environment (G×E) interaction, testing the interaction between depression-PGRS and traumatic events in adulthood on all phenotypes that are significantly associated with depression-PGRS at a minimum of four p thresholds in the discovery and replication datasets. Beta values (standardised regression coefficients), two-sided, uncorrected p-values and FDR-corrected p-values are both reported for the linear regression.

Title: Supplementary Data 10.

Description: Results for gene by environment (G×E) interaction, testing the interaction between depression-PGRS and traumatic events in childhood on all phenotypes that are significantly associated with depression-PGRS at a minimum of four p thresholds in the discovery and replication datasets. Beta values (standardised regression coefficients), two-sided, uncorrected p-values and FDR-corrected p-values are both reported for the linear regression.

Title: Supplementary Data 11.

Description: Results for gene by environment (G×E) interaction, testing the interaction between depression-PGRS and current stressful life events on all phenotypes that are significantly associated with depression-PGRS at a minimum of four p thresholds in the discovery and replication datasets. Beta values (standardised regression coefficients), two-sided, uncorrected p-values and FDR-corrected p-values are both reported for the linear regression.

Title: Supplementary Data 12.
Description: Results for gene by environment (G×E) interaction, testing the interaction between depression-PGRS and household income on all phenotypes that are significantly associated with depression-PGRS at a minimum of four p thresholds in the discovery and replication datasets. Beta values (standardised regression coefficients), two-sided, uncorrected p-values and FDR-corrected p-values are both reported for the linear regression.

Title: Supplementary Data 13.
Description: Results for gene by environment (G×E) interaction, testing the interaction between depression-PGRS and Townsend Index tertiles on all phenotypes that are significantly associated with depression-PGRS at a minimum of four p thresholds in the discovery and replication datasets. Beta values (standardised regression coefficients), two-sided, uncorrected p-values and FDR-corrected p-values are both reported for the linear regression.

Title: Supplementary Data 14.
Description: Results for gene by environment (G×E) interaction, testing the interaction between depression-PGRS and sex on all phenotypes that are significantly associated with depression-PGRS at a minimum of four p thresholds in the discovery and replication datasets. Beta values (standardised regression coefficients), two-sided, uncorrected p-values and FDR-corrected p-values are both reported for the linear regression. This additional result is shown here in order to illustrate possible sex difference in the polygenic architecture of depression that may contribute to the wide-spread sex difference in variables relevant to depression and the brain. However, no significant interaction was found ($p_{FDR} > 0.896$).
